# Cytosolic peptides encoding Ca_V_1 C-termini downregulate the calcium channel activity-neuritogenesis coupling

**DOI:** 10.1038/s42003-022-03438-1

**Published:** 2022-05-19

**Authors:** Yaxiong Yang, Zhen Yu, Jinli Geng, Min Liu, Nan Liu, Ping Li, Weili Hong, Shuhua Yue, He Jiang, Haiyan Ge, Feng Qian, Wei Xiong, Ping Wang, Sen Song, Xiaomei Li, Yubo Fan, Xiaodong Liu

**Affiliations:** 1 Key Laboratory for Biomechanics and Mechanobiology of Ministry of Education, Beijing Advanced Innovation Center for Biomedical Engineering, School of Biological Science and Medical Engineering, School of Engineering Medicine, Beihang University, Beijing, 100083 China; 2 X-Laboratory for Ion-Channel Engineering, Beihang University, Beijing, 100083 China; 3 School of Medicine, Tsinghua University, Beijing, 100084 China; 4 Center for Life Sciences, School of Life Sciences, Yunnan University, Kunming, 650091 China; 5 School of Pharmaceutical Sciences, Tsinghua University, Beijing, 100084 China; 6 School of Life Sciences, Tsinghua University, Beijing, 100084 China; 7 Laboratory for Biomedical Engineering of Ministry of Education, Zhejiang University, Hangzhou, 310027 China

**Keywords:** Calcium channels, Peptides

## Abstract

L-type Ca^2+^ (Ca_V_1) channels transduce channel activities into nuclear signals critical to neuritogenesis. Also, standalone peptides encoded by Ca_V_1 DCT (distal carboxyl-terminus) act as nuclear transcription factors reportedly promoting neuritogenesis. Here, by focusing on exemplary Ca_V_1.3 and cortical neurons under basal conditions, we discover that cytosolic DCT peptides downregulate neurite outgrowth by the interactions with Ca_V_1’s apo-calmodulin binding motif. Distinct from nuclear DCT, various cytosolic peptides exert a gradient of inhibitory effects on Ca^2+^ influx via Ca_V_1 channels and neurite extension and arborization, and also the intermediate events including CREB activation and c-Fos expression. The inhibition efficacies of DCT are quantitatively correlated with its binding affinities. Meanwhile, cytosolic inhibition tends to facilitate neuritogenesis indirectly by favoring Ca^2+^-sensitive nuclear retention of DCT. In summary, DCT peptides as a class of Ca_V_1 inhibitors specifically regulate the channel activity-neuritogenesis coupling in a variant-, affinity-, and localization-dependent manner.

## Abstract (web summary)

When present in the cytosol, a variety of peptides encoded by the distal carboxyl-termini of the L-type Ca_V_1 calcium channels downregulate channel activity and neurite outgrowth.

## Introduction

Voltage-gated Ca^2+^ channels (Ca_V_) are closely involved in diverse pathophysiological processes, by generating Ca^2+^ signals in response to membrane potentials^1,2^. L-type Ca^2+^ channels (Ca_V_1) from the Ca_V_ family, Ca_V_1.2 and Ca_V_1.3 in particular, are widely expressed in human tissues and organs including the nervous system^3^. Among its multiple roles, Ca_V_1 channels mediate the signaling cascade known as excitation-transcription coupling, which transduces cellular stimuli into nuclear signals to regulate transcription of essential genes manifested into the growth conditions of neurites, which constitutes Ca_V_1-dependent excitation-neuritogenesis coupling^4–7^. Notably, such coupling between Ca_V_1 channels and neuritogenesis signaling is also functional even under basal conditions since Ca^2+^ channels are still active, e.g., to mediate the slow calcium oscillations^8,9^. That says, the essential factor to couple the downstreams is the activities of Ca_V_1 channels, either at low (basal) or high (excited) levels, which explains the up- or downregulations of Ca_V_1-mediated signaling and neuritogenesis upon high K^+^ stimulation and other perturbations^10,11^. Both Ca_V_1 functions and neurite development are closely involved in learning and memory, and a broad spectrum of mental disorders^12,13^. The cascade linking Ca_V_1 to neuritogenesis is coordinated by a number of key Ca^2+^-signaling proteins including calmodulin (CaM), Ca^2+^/CaM-dependent Kinase II (CaMKII), and calcineurin, to accomplish: Ca_V_1 channel gating and Ca^2+^ influx, cytonuclear translocation of key molecules CaM and CaMKII, activation of nuclear transcription factors such as CREB (cAMP-response element-binding protein) and NFAT (nuclear factor of activated T-cells), expression of critical genes including c-Fos, and ultimately branching and elongation of neurites^10,14–18^. In parallel, the peptide fragments encoded by distal carboxyl-terminus (DCT) of Ca_V_1 could also act as transcription factors in the nucleus that directly regulate gene transcription and expression, to promote neurite outgrowth^19^.

The effects of DCT as the key domain of Ca_V_1 have been well characterized. By competing with apoCaM (Ca^2+^-free calmodulin), intramolecular DCT autonomously binds the canonical CaM-binding motif (the preIQ-IQ domain of Ca_V_1), causing concurrent effects on channel gating, i.e., weaker Ca^2+^-dependent inactivation and reduced voltage-gated activation^20–23^. Chemical-induced dimerization of the proximal- and distal-DCT subdomains demonstrated that the reduction of Ca^2+^ influx is solely due to acute binding of DCT, ruling out other potential mechanisms of action^21,24,25^. Effects of DCT on Ca_V_1 are in direct opposition to apoCaM, consistent with a mechanism of strict competition between DCT and apoCaM^20,23^. As summarized by the term CMI (C-terminus mediated inhibition)^24^, DCT is able to produce multifaceted and coherent effects, including competitive binding against apoCaM, reduction of Ca^2+^ influx, and concurrent attenuation of inactivation and activation.

Besides intramolecular DCT inhibition, standalone DCT peptides have also been reported to bind and inhibit Ca_V_1 channels^20–22,26–31^. We postulated that DCT peptides through a mechanism of intermolecular CMI should attenuate Ca^2+^/Ca_V_1 signaling to the nucleus. In support, some Ca_V_1 inhibitors such as dihydropyridine (DHP) do exert inhibitory effects on Ca_V_1-dependent signaling and neuritogenesis^10,11,32^. We pursued the above hypothetical inhibition on Ca_V_1-dependent neuritogenesis by standalone DCT peptides, which, however, would be in direct contradiction to neuritogenic DCT effects as demonstrated by CCAT (Calcium Channel Associated Transcription regulator, encoded by Ca_V_1.2 DCT)^19^, thus encountered with an immediate dilemma. In this work, we are motivated by such peptide CMI that supposedly downregulates the Ca_V_1-neuritogenesis coupling through a unique mechanism of action: DCT peptides bind Ca_V_1 at its CaM-binding motif.

DCT peptides have been found endogenously in native cells under physiological conditions^33^. These peptides could result from distinct production mechanisms in various cell types, and may have different lengths or compositions, but are all encoded by DCT fragments of high homology across the Ca_V_1 family (Supplementary Fig. 1 and Supplementary Table 1). In skeletal muscle cells, Ca_V_1.1-encoded peptides of ~30-40 kDa are produced by proteolysis^34–36^, named as CCT_S_ (Cleaved Carboxyl-Terminal fragment from Ca_V_1.1 pore-forming subunit α_1S_; and CCT_C_ and CCT_D_ from α_1C_ and α_1D_, respectively). Similarly, CCT_C_ is cleaved from Ca_V_1.2 in neurons^37^ or cardiac myocytes^38,39^. Presumably by Ca_V_1.3 cleavage, CCT_D_ of ~40 kDa was found in cardiac myocytes^40^. Besides proteolytic cleavage, DCT peptides could also be generated by bicistronic mechanisms via exonic promoters for direct translation^19,41–43^, e.g., CCAT_C_ of ~60 kDa or more. In summary, two types of DCT peptides (~40 kDa and ~60 kDa) have been evidenced from diverse preparations^33^, alongside with a shorter peptide of ~15 kDa in neurons (encoded by the last ~100 a.a. at the C-terminal end of Ca_V_1.2)^41^. Substantial discrepancies exist among various peptides in sequences, mechanisms of production, specificity to cell types, and effects on channels or cells. In this work, we undertook the task to clarify the actual roles of DCT-encoded peptides, focusing on the hypothesis that these peptides of diverse forms essentially share the same principle: affinity-dependent binding of Ca_V_1 channels to downregulate Ca_V_1 activities and channel activity-dependent neuritogenesis.

## Results

### Hints on DCT peptide inhibition of Ca_V_1 channels and neuritogenesis

We first conducted sequence alignment for homologous DCT domains across Ca_V_1.1-1.4, containing the major fragments of proximal C-terminal regulatory domain (PCRD), nuclear retention domain (NRD) and distal C-terminal regulatory domain (DCRD) (Supplementary Fig. 1 and Supplementary Table 1). The NRD motif is an indispensable region for Ca^2+^-dependent nuclear export as demonstrated in CCAT_C_^19^ and CCT_D_^40^. The PCRD and DCRD cooperate to compete with apoCaM for binding the IQ motif of Ca_V_1 as the molecular basis of CMI, where the DCRD plays a dominant role compared with the PCRD^22,24^. Two transcription activation domains are localized in the PCRD-NRD junction and the DCRD motif^19,41^, respectively. Based on these and earlier analyses, the representative peptides of three major categories have been focused on, including 1) DCT peptides (~60 kDa) via bicistronic transcription, such as CCAT_C_ that contains the entire DCT; 2) CCT_C_ or CCT_D_ (~40 kDa) from posttranslational cleavage lacking the PCRD domain but still incorporating the majority of DCT (from NRD to DCRD); 3) the short peptide of DCRD (~15 kDa) that is sufficient to modulate Ca_V_1 gating, although its physiological relevance is relatively less established.

**Fig. 1:**
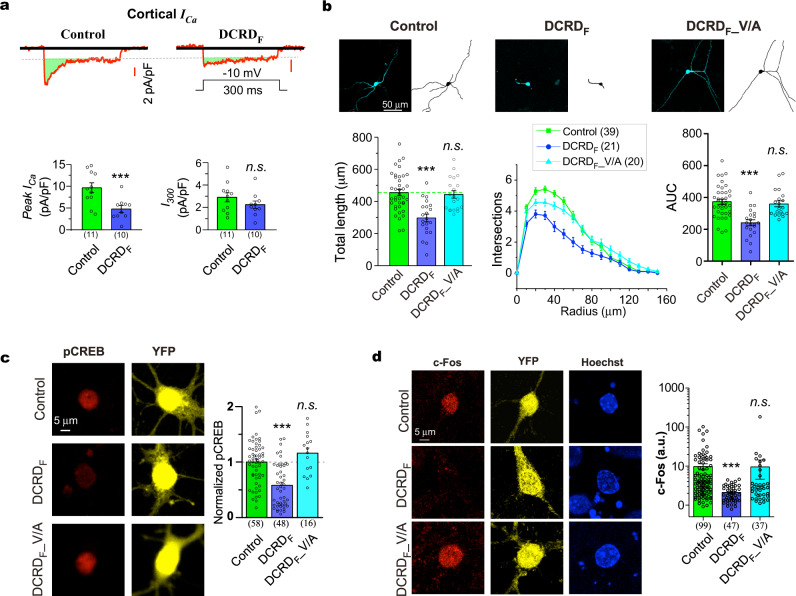
DCRD_F_ effects on Ca_V_1.3 gating and neuritogenesis signaling in cortical neurons. **a** Inhibition of endogenous Ca_V_1.3 currents by DCRD_F_ peptides. Cultured neurons were treated with the cocktail recipe to isolate Ca_V_1.3 Ca^2+^ current (details in Supplementary Fig. 2). Exemplary Ca^2+^ current (upper, scale bar in red) was elicited by the standard protocol of voltage step (300 ms; -10 mV). Potential inhibitory effects on channel functions were evaluated by the indices of peak and steady-state current amplitudes (pA/pF, bottom). **b** Effects on neuronal morphology. Based on the confocal fluorescent images of peptide-expressing cortical neurons (upper row), neurite tracing for each neuron was performed (middle row), total neurite length and Sholl analyses summary (bottom row) were compared among the three groups: YFP, YFP-DCRD_F_ and YFP-DCRD_F__V/A. Sholl analyses are routinely accompanied by the quantitative index of AUC (area under the curve, lower right). **c** Effects of DCRD_F_ peptides on pCREB signals. Cortical neurons were transfected with YFP, YFP-DCRD_F_ or YFP-DCRD_F__V/A (loss-of-function mutant), respectively. The pCREB signals were evaluated by immunofluorescence. Red and yellow fluorescence in confocal images represent pCREB signals and overexpressed YFP, respectively. pCREB signals were normalized over the YFP control group. **d** Effects on c-Fos signals. Cortical neurons expressing YFP, YFP-DCRD_F_ or YFP-DCRD_F__V/A were stained with c-Fos antibody (in red). Fluorescent intensities of c-Fos signals in the nuclei were summarized. Student’s *t*-test (**a**), one-way ANOVA followed by Bonferroni for post hoc tests (**b, c**) and Kruskal-Wallis and Dunn’s non-parametric test (**d**, non-normal distribution, checked by D’Agostino & Pearson omnibus normality test) were used (***p < 0.001; *n.s*., not significant, *p* > 0.05). Values are represented as mean ± SEM.

As proof of principle, the DCRD_F_ was overexpressed in cortical neurons, considering that this short peptide encoded by the last ~100 a.a. of Ca_V_1.4 DCT has been thoroughly characterized for its strong competition with apoCaM to bind onto the channel^20,24,27,31^. By the cocktail treatments of cultured cortical neurons, the relative contribution of Ca_V_1.3 channels to Ca_V_1 currents was evaluated (see Methods and Supplementary Fig. 2 for details). Based on the patch-clamp recordings by a voltage ramp and the representative step of −10 mV, Ca_V_1.3 made a significant contribution to the total Ca_V_1 currents in cortical neurons (~50%), in agreement with the previous reports suggesting that both Ca_V_1.3 and Ca_V_1.2 are critical to Ca_V_1 signaling in cortical neurons and hippocampal neurons^44–48^. With the full cocktail recipe, Ca^2+^ currents mainly mediate by Ca_V_1.3 were isolated and recorded to examine the effects of DCRD_F_ peptides (Fig. 1a). The DCRD_F_ potently attenuated cortical Ca_V_1.3 currents at the peak; meanwhile, the steady-state amplitude (measured at 300 ms) was nearly unchanged. Such characteristic effects on native Ca_V_1.3 channels are highly consistent with the CMI modulation of recombinant Ca_V_1.3 channels^24^, where reduction of Ca^2+^ influx is ensured by concurrent attenuation of activation and inactivation evidenced from both acute and long-term effects. We then examined the hypothetical role of DCT inhibition on Ca_V_1-dependent neuritogenesis signaling. As expected, the DCRD_F_ peptide caused a significant reduction in neurite outgrowth and branching of cortical neurons under basal conditions, as measured by the total length and complexity (Sholl analysis) respectively (Fig. 1b). In contrast, the mutant peptide DCRD_F__V/A (V/A denotes V41A, a loss-of-function mutation) produced no damage on neurite outgrowth of cortical neurons overexpressing DCRD_F_.

We further checked the major signals along the well-established cascade, including the phosphorylation of a key transcription factor CREB^49,50^. Ca_V_1 would be the major path of Ca^2+^ entry preferred by downstream CaMKII/CREB signaling over Ca_V_2^11^, DCRD_F_ peptides strongly attenuated pCREB signals (immunostaining of phosphorylated CREB or pCREB, Fig. 1c). In contrast, pCREB exhibited no difference between the control neurons and the mutant group DCRD_F__V/A^20,24^. Furthermore, the expression level of c-Fos, one of the classical immediate early genes driven by pCREB^15,16^, was significantly reduced by DCRD_F_ but not DCRD_F__V/A (Fig. 1d). Additional stimulation to enhance membrane excitation or channel activation is expected to provide higher dynamic ranges, although the Ca_V_1 channel activity-neuritogenesis coupling should function similarly in cortical neurons of either conditions (basal or excited). Indeed, when the neurons were stimulated by 40 mM K^+^, similar results from WT and mutant DCRD_F_ were obtained when we reexamined the peptide effects on pCREB signals (Supplementary Fig. 3a). In addition, the enhanced signals by high K^+^ stimulation provided the opportunity to capture the potent inhibition of DCRD_F_ on the translocation of CaM (from Cytosol to Nucleus, defined by N/C ratio, Supplementary Fig. 3b), another key event along the Ca_V_1-triggered signaling pathway^49,50^. Here, the observed effects arise from DCRD_F_ inhibition on Ca_V_1 channels which can be well represented by Ca_V_1.3, especially in cortical neurons under basal conditions.

### CMI effects on recombinant Ca_V_1.3 channels by various DCT peptides

**Fig. 2:**
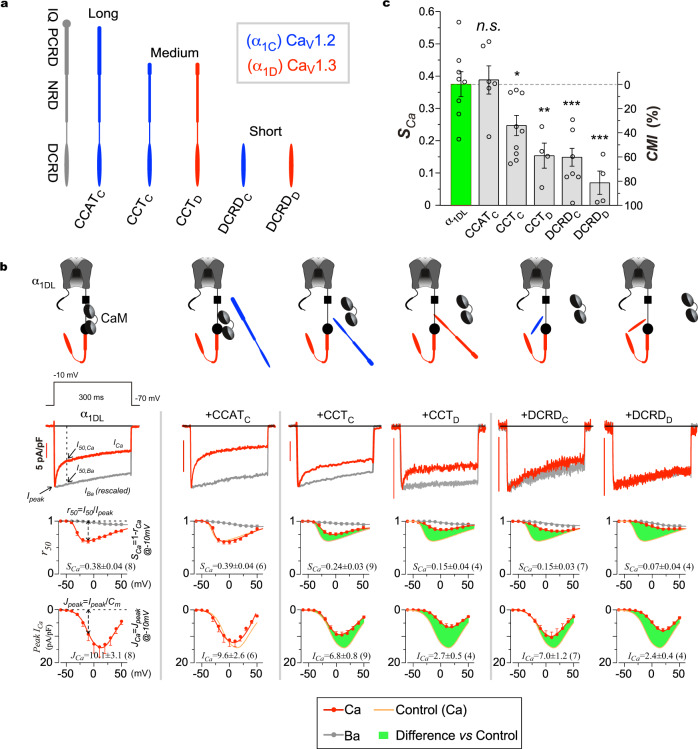
Inhibition of recombinant Ca_V_1.3 channels by representative DCT peptides. **a** Illustration of serial representative peptides encoded from DCT domains of Ca_V_1.2 (blue) and Ca_V_1.3 (red). The long-form peptides (~60 kDa) represented by CCAT_C_ contain the complete set of motifs including PCRD, NRD and DCRD. The medium-form (~40 kDa) representatives CCT_C_ and CCT_D_ start from the very end of the PCRD till the end of the DCRD thus containing both the NRD and the DCRD. The short-form peptides (~15 kDa) are represented here by the peptides DCRD_C_ and DCRD_D_. **b** CMI effects of representative variants on Ca_V_1.3 (α_1DL_) in HEK293 cells. As illustrated by the control group (left column), exemplary Ca^2+^ current (trace with scale bar, red) and Ba^2+^ current (rescaled to Ca^2+^ current at the peaks, gray) were elicited by voltage step at −10 mV (top traces). The next two rows show the profiles of inactivation and activation respectively, with *r*_*50*_ (ration between current amplitudes at 50 ms and the peak) for inactivation and *I*_*peak*_ (Ca^2+^ current) for activation across the full range of membrane potentials (*V*). Based on Ca^2+^ currents at −10 mV, *S*_*Ca*_ (in fraction, 1-*r*_*50*_) and *I*_*Ca*_ (in pA/pF, *I*_*peak*_) serve as the major indices for inactivation and activation, respectively. Five representative peptides of CCAT_C_, CCT_C_, CCT_D_, DCRD_C_ and DCRD_D_ (from left to right) as in (**a**) are compared with α_1DL_ control (the leftmost column) for their inactivation and activation profiles (lines in orange indicate the control). Green areas are to highlight peptide effects. **c** Statistical summary of the extent of Ca^2+^-dependent inactivation (*S*_*Ca*_) and peptide’s CMI potency (*CMI* in percentage). To evaluate the attenuation of DCT peptide on inactivation (*S*_*Ca*_), CMI potency is defined as the change in Ca^2+^-dependent inactivation (*S*_*Ca*_): (*S*_*Ca,Control*_ − *S*_*Ca,Peptide*_)/*S*_*Ca,Control*_, which is equivalent to fractional change in apoCaM-bound channels (*ΔF*_*CaM*_) before (*F*_*CaM*_) and after DCT peptide’s competition. Thus, *CMI* essentially indicates what percentage of apoCaM-bound channels are converted to peptide-bound channels (*f*_*Peptide*_). See Eqs. E2 and E3 in Methods for more details. One-way ANOVA followed by Dunnett for post hoc tests were used for (**c**): *p < 0.05; **p < 0.01; ***p < 0.001. Values are represented as mean ± SEM.

Encouraged by the results that DCT peptides downregulated cortical Ca_V_1 channel-dependent transcription and neuritogenesis, we proceeded further with the recombinant Ca_V_1.3 channels for the details on DCT effects. We chose five variants encoded by DCT of Ca_V_1.2 or Ca_V_1.3 which represent the native forms of DCT peptides: one long-form variant CCAT_C_ (~60 kDa), two medium-form variants CCT_C_ and CCT_D_ (~40 kDa), and two short-form variants DCRD_C_ and DCRD_D_ (~15 kDa) (Fig. 2a). To quantify their effects on the gating of full-length Ca_V_1.3 (α_1DL_) channels, the two major indices were routinely examined: inactivation (the strength of Ca^2+^-dependent inactivation, *S*_*Ca*_) and activation (the peak amplitude of Ca^2+^ current, *I*_*Ca*_)^24^ (Fig. 2b, left column). Firstly, consistent with the previous report^27^, the DCRD_F_ peptides generated characteristic CMI effects: concurrent attenuation of both inactivation and activation, as illustrated by the altered profiles (the green shades to illustrate the actual attenuation) (Supplementary Fig. 4a). In direct contrast, DCRD_F__V/A did not cause any appreciable change in gating indices (*S*_*Ca*_ or *I*_*Ca*_). CMI effects on activation were also evidenced from Ba^2+^ currents which were significantly inhibited by DCRD_F_ but not by DCRD_F__V/A (Supplementary Fig. 4b). Notably, demonstrated by voltage-dependent steady-state (at 300 ms) currents, DCRD_F_ and DCRD_F__V/A peptides are essentially indistinguishable from the α_1DL_ control, supporting the notion that intermolecular CMI (by standalone DCT peptides) shares similar mechanisms with intramolecular CMI (by the DCT motif covalently-linked to the channel), supposedly in an acute manner as previously proved^24^.

Following the initial evidence from the Ca_V_1.4 DCRD_F_, we performed a systematic comparison for the five representative peptides (Fig. 2b), each of which was co-expressed with Ca_V_1.3 channels. To better quantify DCT effects, the inhibition potency (*CMI*, in percentage) is defined as the normalized fraction of channels that switch from apoCaM-bound to DCT-bound, which can be directly calculated from the inactivation parameter *S*_*Ca*_ before and after peptide inhibition (Eqs. E2 and E3 in Methods). *CMI* is inversely proportional to *S*_*Ca*_, therefore strong DCT such as DCRD_F_ should have higher *CMI* than weaker DCT such as DCRD_F__V/A.

By YFP fluorescence intensities, individual cells expressing YFP-tagged DCT were scrutinized to ensure that the expression levels of these peptides were at the comparable (high) levels. DCT peptides of DCRD_C_, CCT_C_, DCRD_D_ and CCT_D_ clearly produced CMI effects of substantial potency, except that CCAT_C_ only slightly attenuated Ca_V_1.3 gating (weak *CMI*) (Fig. 2c). Electrophysiological profiling of peptide CMI lays the foundation for our subsequent investigations into the effects of DCT peptides on Ca_V_1-dependent neuritogenesis. CCAT_C_ exhibited rather weak (insignificant) effects on channel gating, which appears to agree with the earlier report where the long DCT_C_ was found to have no effect on Ca^2+^-dependent inactivation of Ca_V_1.2^22^. However, CCT_C_, the shorter motif encoded by a portion of CCAT_C_, is capable of strong CMI. Also, the key segment DCRD_C_ has the capability to attenuate Ca^2+^-dependent inactivation. In this work, one of our aims is to clarify the above discrepancy regarding various DCT_C_ peptides. In fact, all the four DCT domains across the Ca_V_1 family are homologous (including the DCRD domains), suggesting high similarities in their functional roles (Supplementary Fig. 1). Taking the DCRD_F_ as the exemplar, its core segment was further narrowed down to DCDR_F__17-66 (the residues between S17 and L66) well conserved among Ca_V_1.2-1.4 (but not Ca_V_1.1), which may account for the potent inhibition observed from DCRD_C_, DCRD_D_ or DCRD_F_ peptides (Supplementary Fig. 5).

### Ca_V_1 channels and neuritogenesis are inhibited by cytosolic DCT peptides

**Fig. 3:**
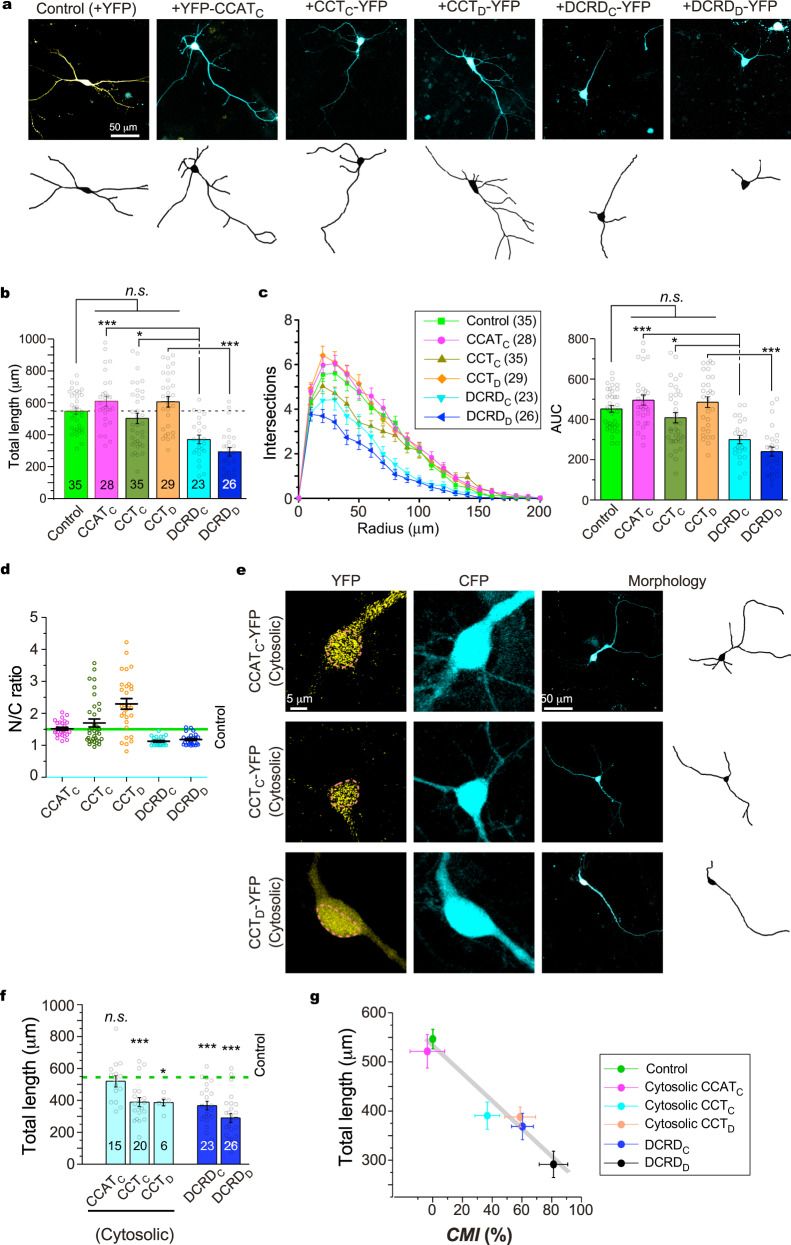
Cytosolic DCT peptides inhibit neurite outgrowth of cortical neurons. **a** Fluorescence images of cortical neurons (DIV-7) expressing CFP and DCT variants tagged with YFP. Representative confocal images (upper row, merged CFP in cyan with YFP in yellow) and corresponding neurite tracing (bottom row) are shown for YFP control, CCAT_C_, CCT_C_, CCT_D_, DCRD_C_ and DCRD_D_, respectively. **b**, **c** Statistical summary of total neurite length (**b**) and Sholl analyses (**c**). **d** Statistical summary of N/C ratio for all the five groups of DCT peptides. Horizontal line indicates N/C ratio of YFP as the criteria (for DCT distribution) to assign each neuron to either the nuclear subgroup (N/C ratio>1.5) or the cytosolic subgroup (N/C ratio<1.5). **e** Representative confocal images, neurite tracings and detailed cytonuclear distribution are shown for neurons with cytosolic CCAT_C_, CCT_C_ and CCT_D_, respectively. The envelope of the nucleus is highlighted by live-cell Hoechst 33342 staining (dotted lines). **f** Total length for the neurons from the cytosolic groups: peptides of CCAT_C_, CCT_C_ and CCT_D_, and also the groups of DCRD_C_ and DCRD_D_ peptides. **g** A potential correlation between CMI values (adopted from Fig. 2c) and total neurite length (*R*^*2*^ = 0.93). One-way ANOVA followed by Bonferroni or Dunnett for post hoc tests were used for (**b**, **c**) and (**f**), respectively (*p < 0.05; **p < 0.01; ***p < 0.001). Values are represented as mean ± SEM.

In the context of Ca_V_1-dependent neuritogenesis, modulation of Ca_V_1 channels would make changes in the growth of neurites, provided that the channel activity-transcription coupling is coherently regulated. Based on the potent inhibition by DCRD_F_ (Fig. 1), we pursued the hypothesis further that the DCT peptides of native forms may induce inhibitory effects in accordance with CMI potency (Fig. 2c). Since the signals or events at all the checkpoints (Ca_V_1 gating, Ca^2+^ influx, CaM translocation, pCREB, c-Fos and neuritogenesis) were consistently attenuated by DCRD_F_ in cortical neurons (Fig. 1), Ca_V_1 gating and neurite growth, the major input and output respectively, were selected as the two major checkpoints to represent the full cascade of channel activity-neuritogenesis coupling. By overexpressing DCT peptides in cortical neurons with careful scrutinization of cellular fluorescence as in electrophysiology (Fig. 2), the potential CMI on neuritogenesis was examined for the representative peptides of CCAT_C_, CCT_C_, and CCT_D_ (long and medium forms), along with the peptides DCRD_C_ and DCRD_D_ (short form). To our surprise, statistically none of CCAT_C_, CCT_C_, and CCT_D_ exhibited any significant effect on neurite length and branching even for ensured overexpression (Fig. 3a–c). Meanwhile, resembling DCRD_F_ inhibition, DCRD_C_ and DCRD_D_ peptides induced significant neurite retractions. Since functional Ca_V_1 channels are located at the plasma membrane, CMI effects by DCRD peptides should take place in the cytosol. We then examined the cytosolic-nuclear distribution for each peptide variant, indexed by its N/C ratio (Fig. 3d). On average, the N/C ratio values for DCRD_C_ and DCRD_D_ fell below the control level (YFP, with N/C ratio ~1.5)^51^, showing a pattern of cytosolic distribution; in contrast, the peptides CCAT_C_, CCT_C_, and CCT_D_ were more distributed into the nucleus (N/C ratio>1.5). For each variant, by applying N/C ratio criteria (cut-off value of 1.5) the neurons could be divided into two distinct (cytosolic versus nuclear) subgroups. For the cytosolic subgroup, similar to DCRD_C_ and DCRD_D_, cytosolic CCT_C_ and CCT_D_ significantly attenuated neurite outgrowth (Fig. 3e, f). Notably, although cytosolic CCAT_C_ appeared to have a tendency of attenuation, its actual effects on neurites turned out to be rather mild with no statistical significance, consistent with its weak CMI on Ca_V_1.3 gating. We then postulated that cytosolic DCT peptides would downregulate the Ca_V_1-dependent neuritogenesis. In support, for the five representative peptides we tested in cortical neurons, an inverse correlation appeared to exist between CMI potency of cytosolic peptides and neurite length (Fig. 3g). Hence, it is likely that the DCT peptides present in the cytosol share the same mechanisms with DCRD_F_ to inhibit Ca_V_1 gating and signaling (Fig. 1).

### Both PCRD and DCRD tune CMI potency of DCT variants

**Fig. 4:**
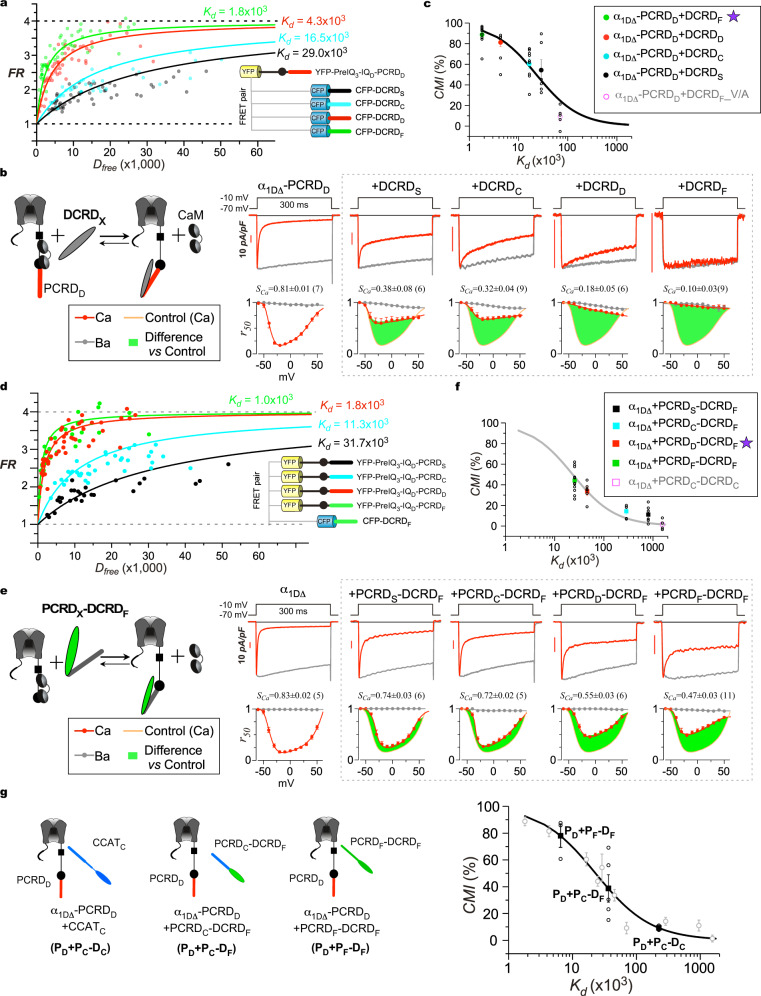
Both PCRD and DCRD play important roles in CMI unveiled by DCT variants. **a** Binding curves for the interactions between key channel motifs and DCRD peptides were quantified by 2-hybrid 3-cube FRET, for each pair between CFP-DCRD_X_ from Ca_V_1 (X = S, D, C and F) and YFP-preIQ_3_-IQ_D_-PCRD_D_. *D*_*free*_ and *FR* represent free donor concentration and FRET ratio, respectively. The binding affinity *K*_*d*_ for each pair was achieved by iterative fitting. In particular, the pair of PCDR_D_ and DCRD_F_ serves as the major reference for subsequent analyses. **b** Comparison of CMI potency among DCRD_X_ peptides. As in the cartoon illustration of peptide CMI (top), the peptide DCRD_X_ could coordinate with the IQ_D_ and PCRD_D_ motifs on α_1DΔ_-PCRD_D_ to compete apoCaM off the channel. Ca^2+^ trace exemplars and inactivation profiles are shown for α_1DΔ_-PCRD_D_ alone or with different DCRD_X_ isoforms. **c** Relationship between *K*_*d*_ and *CMI* for DCRD_X_ peptides. Four peptides directly from Ca_V_1.1-Ca_V_1.4, plus one additional mutant peptide DCRD_F__V/A (Supplementary Fig. 6). The relationship between *K*_*d*_ and *CMI* for DCRD_X_ peptides was fit by Eq. E4 (see Methods). **d** Similar to DCRD measurements (**a**), PCRD_X_ peptides across Ca_V_1 family (X = S, D, C, and F) were also quantified by FRET for the interactions between YFP-preIQ_3_-IQ_D_-PCRD_X_ and CFP-DCRD_F_. The two sets of measurements share the common pair PCRD_D_ and DCRD_F_ as the major index. See *K*_*d*_ values of PCRD_X_-DCRD_X_ in Supplementary Fig. 7. **e** Comparison of CMI potency among PCRD_X_-DCRD_F_ peptides. As illustrated by the cartoon (top), PCRD_X_-DCRD_F_ could form the complex with IQ_D_ of α_1DΔ_ to compete with apoCaM. In similar fashion, Ca^2+^ trace exemplars and inactivation profiles are shown for α_1DΔ_ alone or with long-form peptides PCRD_X_-DCRD_F_. **f** Further support for the proposed *CMI-K*_*d*_ correlation. Additional data points (*CMI* and *K*_*d*_) of PCRD_X_-PCRD_F_ (**e**) and PCRD_C_-DCRD_C_ (Supplementary Fig. 8) are superimposed onto the ligand-binding curve from (**c**). **g** The tuning curve of *K*_*d*_-dependent *CMI* with the summary of peptides. For compound effects of long-form DCT peptides on α_1DΔ_-PCRD_D_, three more data points (black squares) were added onto the tuning curve, based on *CMI* measurements and *K*_*d*_ estimations for P_D_+P_C_-D_C_ (or P_D_+CCAT_C_), P_D_+P_C_-D_F_ and P_D_+P_F_-D_F_ (Supplementary Fig. 9). Values are represented as mean ± SEM.

No structural information of DCT is available thus far^52–54^. To gain further insights into the mechanisms underlying DCT effects, we firstly focused on CCAT_C_, unexpectedly exerting rather mild inhibition on Ca_V_1.3 channels and cortical neurons (Fig. 3g). In contrast to CCAT_C_, the shorter peptides of DCRD_C_ and CCT_C_ both encoded by Ca_V_1.2 DCT have strong CMI, suggesting a self-limiting mechanism within the longer CCAT_C_. Moreover, Ca_V_1.2 has been considered to have the same level of Ca^2+^-dependent inactivation with or without its DCT domain^22,55^, inconsistent with strong CMI of DCRD_C_ in our experiments (Fig. 2). To resolve these discrepancies, we performed systematic analysis with the representative DCT peptide variants. By utilizing 2-hybrid 3-cube FRET (Förster resonance energy transfer), a quantitative imaging assay for protein-protein interactions in live cells^24,56^, the capabilities of DCRD peptides to bind the channel were quantified by dose-dependent binding (*FR-D*_*free*_) curves (Fig. 4a). Following the convention, we employed the (effective) dissociation equilibrium constant (*K*_*d*_, units in fluorescence intensities through the donor cube) as the index of binding affinities. Utilizing CFP-tagged DCRD_X_ peptides (X = S, D, C, and F representing Ca_V_1.1-1.4) and YFP-tagged preIQ_3_-IQ_D_-PCRD_D_ (CaM-binding motif of Ca_V_1.3) as the FRET pairs, a series of binding curves were achieved by iterative fitting processes (Fig. 4a). Among a gradient of *K*_*d*_ values from the four pairs of binding, DCRD_F_ encoded by Ca_V_1.4 resulted in the strongest affinity (*K*_*d*_ = 1.8 × 10^3^), followed by the peptides DCRD_D_ (*K*_*d*_ = 4.3 × 10^3^), DCRD_C_ (*K*_*d*_ = 16.5 × 10^3^) and DCRD_S_ (*K*_*d*_ = 29.0 × 10^3^).

In parallel with FRET binding analyses, the whole-cell electrophysiology was performed for functional characterizations. DCRD_X_ peptides were overexpressed with α_1DΔ_-PCRD_D_, a channel variant producing ultra-strong Ca^2+^-dependent inactivation (due to lacking the critical DCRD domain) thus providing an ample dynamic range to evaluate CMI effects. All of the four DCRD_X_ peptides caused inhibitory effects of different potency on α_1DΔ_-PCRD_D_ channels, illustrated by their inactivation (*S*_*Ca*_) profiles (Fig. 4b). The classic ligand binding (Langmuir isotherm) equation (see Methods: Eq. E4) between inhibition potency *CMI* and binding affinity *K*_*d*_ was utilized to describe the differential effects among the peptide variants (Fig. 4c). For the mutant DCRD_F__V/A, both peptide binding and channel inhibition were severely perturbed by the critical mutation (Supplementary Fig. 6), also agreeing well with the tuning curve of *K*_*d*_-dependent *CMI* (Fig. 4c).

In parallel, the potencies of PCRD_X_ (X = S, D, C and F) were examined with FRET pairs YFP-preIQ_3_-IQ_D_-PCRD_X_ and CFP-DCRD_F_. Similar to DCRD_X_, *K*_*d*_ values were obtained for the PCRD_X_ peptides, unveiling the relative order of strength in binding (starting from the strongest): PCRD_F_, PCRD_D_, PCRD_C_, and PCRD_S_ (Fig. 4d). The difference in *K*_*d*_ between PCRD_C_ (*K*_*d*_ = 11.3 × 10^3^) and PCRD_D_ (*K*_*d*_ = 1.8 × 10^3^) is even more pronounced than that between DCRD_C_ and DCRD_D_ (6.3-fold versus 3.8-fold), suggesting that the rather weak inhibition by DCT_C_ (either as the intramolecular motif or the intermolecular peptide) is mainly attributed to its proximal domain PCRD_C_. Such result is unexpected, since the PCRD motif has been considered to play a much lesser role (than the DCRD motif) in DCT effects. For instance, it has been reported that PCRD is not required for channels inhibition under the low CaM conditions whereas DCRD still remains indispensable to CMI^24^. For fair comparison, the pair of DCRD_F_ and preIQ_3_-IQ_D_-PCRD_D_ is taken as the principle reference (noted as PCRD_D_/DCRD_F_ or its abbreviation P_D_/D_F_) (Supplementary Table 1). All combinations of PCRD_X_/DCRD_X_ (abbreviated as P_X_/D_X_) are summarized to compare their *K*_*d*_ values (Supplementary Fig. 7). Besides experimental values from FRET, *K*_*d*_ for other P_X_/D_X_ combinations can also be roughly estimated according to the values assigned to *P*_*X*_ and *D*_*X*_. For validation purposes, FRET experiments were conducted for P_C_/D_C_ (Ca_V_1.2) and P_S_/D_S_ (Ca_V_1.1) (Supplementary Fig. 8), resulted in rather weak binding affinities (*K*_*d*_), consistent with the predictions from P_X_ and D_X_ (Supplementary Fig. 7).

Similar to DCRD, the functional role of PCRD was also examined, but by co-expressing PCRD_X_-DCRD_F_ (i.e., P_X_-D_F_) with α_1DΔ_ channels. P_S_-D_F_ and P_C_-D_F_ peptides exhibited much weaker inhibition than P_D_-D_F_ and P_F_-D_F_, indicated by less changes in Ca^2+^-dependent inactivation of α_1DΔ_ channels (Fig. 4e). The four peptides of P_X_-D_F_ comply with the same tuning curve of *CMI*-*K*_*d*_ in Fig. 4c, but here with P_X_ as the factor subject to variations (Fig. 4f). In addition, the relatively weak *K*_*d*_ and *CMI* for P_C_-D_C_ (Supplementary Fig. 8) agree well with the tuning curve, as one additional validation for its applicability to P_X_-D_X_ peptides.

In summary, both PCRD and DCRD may underlie the distinct effects of DCT peptides across the Ca_V_1 family^20,22,57,58^. Here, the importance of PCRD is unmasked. The ultra-weak CMI potency of DCT_S_ or DCT_C_ is mainly attributed to its PCRD domain, in that DCRD_S_ and DCRD_C_ are fully capable of strong CMI effects (Fig. 4b). In this context, for CCT_D_, CCT_C_ and CCAT_C_ (here equivalent to DCRD_D_, DCRD_C_, and DCT_C_), the potencies of CMI effects (from strong to weak inhibition) on Ca_V_1 (represented by Ca_V_1.3, Fig. 2c) are expected to be in the same order as their binding affinities: P_D_/D_D_, P_D_/D_C_, and P_C_/D_C_ (from strong to weak binding).

### Compound effects on α_1DL_ channels by long DCT peptides

Although the weak inhibition by the long peptides DCT_C_ or CCAT_C_ could be attributed to its PCRD_C_, it is still unclear why Ca_V_1.3 (the full-length channel containing intramolecular PCRD_D_) is barely regulated by CCAT_C_ peptides. To fully elucidate the mechanism underlying the weak CCAT_C_, we decomposed its effects on α_1DΔ_-PCRD_D_ channels into two scenarios (Fig. 4g). The first component (I) represents the combination of P_D_ from the channel and D_C_ from the peptide, which produces strong inhibitory effects. The second component (II) represents the combination of P_C_ and D_C_ both from the long peptide DCT_C_ (equivalent to CCAT_C_), which has rather weak *CMI* (Supplementary Fig. 9a, b). Overall, CMI potency of CCAT_C_ on α_1DΔ_-PCRD_D_ is expected to fall into the range defined by both components (I and II) corresponding to the upper- and lower-limit respectively. The compound effects of CCAT_C_ resulted in weak CMI potency toward its lower limit, suggesting a dominant role of PCRD_C_ in this particular scenario. For validation purposes, P_C_-D_F_ (PCRD_C_ fused with DCRD_F_) was constructed and applied as an artificial type of DCT peptides (Supplementary Fig. 9c, d). Similar to P_C_-D_C_, the effects of engineered P_C_-D_F_ on α_1DΔ_-P_D_ could also be decomposed into two combinations, where PCRD_C_ compromised the ultrastrong CMI of DCRD_F_ and thus the overall CMI only reached an intermediate level.

Collecting the data from PCRD or DCRD variants (Fig. 4c, f), a tuning curve between CMI potency and binding affinity (*CMI-K*_*d*_) has been established, applicable to a broad scope of channel and peptide variants (Fig. 4g). In principle, for any DCT peptide variants, of either native or engineered and either WT or mutant forms, when applied to Ca_V_1.3 channels (supposedly to Ca_V_1 channels in general), the potency of CMI quantitatively would correlate with the affinity between peptides and channels, which is also a measure of the competition (against apoCaM) introduced by DCT peptides. Based on the tuning curve, *K*_*d*_ values for particular peptides can be estimated from their *CMI* values measured in electrophysiology, which has been demonstrated by P_C_-D_C_ (or CCAT_C_) and P_C_-D_F_, and also by another long peptide P_F_-D_F_ (Supplementary Fig. 9e, f). Furthermore, CCT_S_^36^, hypothetical CCAT_D_ or CCT_F_, and more other variants, can also be evaluated or predicated for the effects on Ca_V_1 channels according to such unified tuning curve of *CMI-K*_*d*_.

### Cytosol/nucleus-dependent effects of DCT peptides reconcile the discrepancy in neurons

**Fig. 5:**
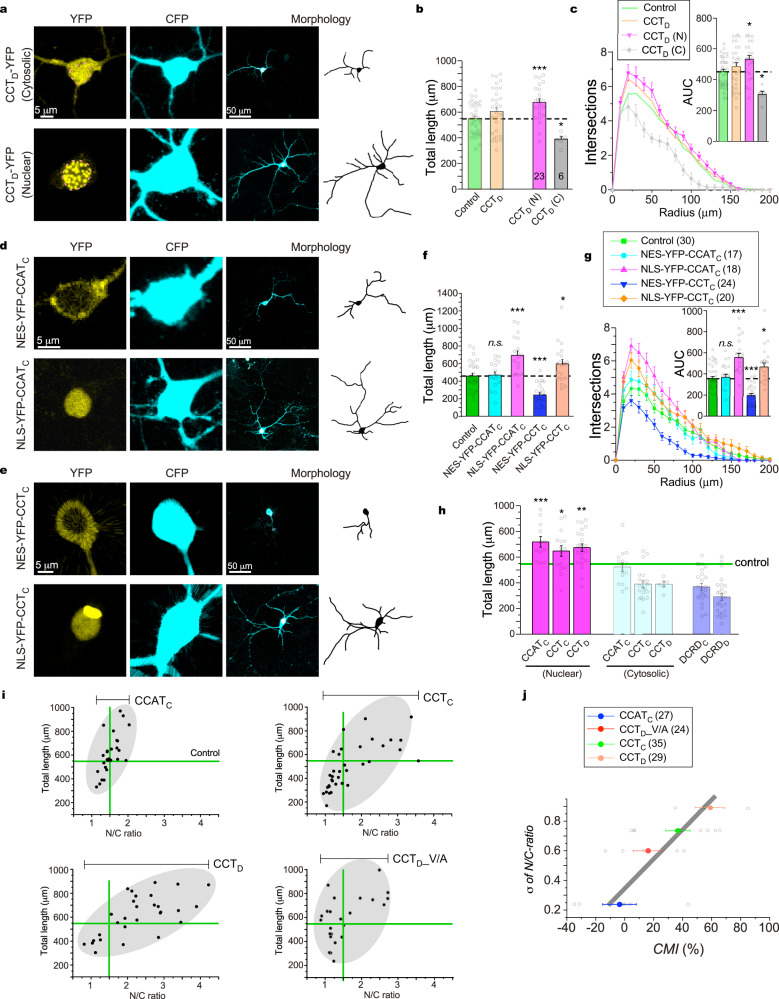
Opposite effects of cytosolic versus nuclear DCT peptides on neurite outgrowth. **a** Cortical neurons with CCT_D_ fragments are grouped into two categories: nuclear *versus* cytosolic, i.e., CCT_D_ (N) and CCT_D_ (C) by the same criteria of N/C ratio as in Fig. 3 (CCT_D_ fluorescence with N/C ratio >1.5 for the nuclear group; and N/C ratio <1.5 for the cytosolic group). **b**, **c** Total neurite length (**b**) and Sholl analyses (**c**) for neuron groups of Control, total CCT_D_, CCT_D_ (C) and CCT_D_ (N). Original data for Control and total CCT_D_ groups are adopted from Fig. 3b-e NES and NLS were fused to N-terminus of YFP-CCAT_C_ (**d**) or YFP-CCT_C_ (**e**) to constrain the distribution of DCT peptides within the cytosol or the nucleus, respectively. **f**, **g** Total neurite length (**f**) and Sholl analyses (**g**) for the groups of Control, NES-YFP-CCAT_C_ versus NLS-YFP-CCAT_C_, and NES-YFP-CCT_C_ versus NLS-YFP-CCT_C_. **h** Total neurite lengths for neurons overexpressing CCAT_C_, CCT_C_ and CCT_D_ peptides are summarized and compared among nuclear versus cytosolic subgroups, together with DCRD_C_ and DCRD_D_ groups (adopted from Fig. 3f). **i** Cyto-nuclear distribution (indexed by N/C ratio) of DCT peptides in correlation with the total neurite length. Horizontal and vertical lines in green represent the control group (YFP expressed in cortical neurons). The range of N/C ratio is indicated on top of the scatter plot for each peptide group of CCAT_C_, CCT_C_, CCT_D_, or the mutant CCT_D__V/A. The grey shades for peptide variants are to illustrate the potential correlation between neurite length and N/C ratio. **j** Correlation between CMI strength and cyto-nuclear distribution. For four peptide variants of CCAT_C_, CCT_C_, CCT_D_ and CCT_D__V/A, standard deviations of N/C ratio values are represented by σ (*N/C ratio*) to quantify spatial dynamics for each peptide variant. *CMI* and σ are highly correlated (linear fit, *R*^*2*^ = 0.90). One-way ANOVA followed by Dunnett for post hoc tests were used for (**b**, **c**), and (**f**–**h**) (*p < 0.05; **p < 0.01; ***p < 0.001). Values are represented as mean ± SEM.

Our data thus far demonstrate that cytosolic DCT peptides negatively regulate neurite outgrowth, intrinsically tuned by *CMI* (channel inhibition) or *K*_*d*_ (peptide binding) in a variant-dependent manner. The apparent contradictions regarding DCT effects (inhibitory versus facilitatory) may simply reflect the differential roles of peptides in the cytosol versus in the nucleus. In order to test this hypothesis, we first revisited the overexpressing CCT_D_ which was widely distributed across the whole cell, featuring a broad range of N/C ratio values (Fig. 3d). Dual directional effects are evident: for the nuclear group CCT_D_ (N) with N/C ratio>1.5, neurite outgrowth was promoted; in contrast, for neurons from the cytosolic group (N/C ratio<1.5) or CCT_D_ (C), neurite outgrowth was significantly reduced similar to DCRD_D_ (Fig. 5a–c). To confirm this result, the short tags of nuclear export signal (NES) and nuclear localization signal (NLS) were fused to the N-terminus of CCAT_C_ or CCT_C_. In doing so, NES-tagged CCAT_C_ and CCT_C_ were predominantly expressed in the cytosol (Fig. 5d, e). In comparison with the minor effects of NES-CCAT_C_, neurite retractions were evidenced from NES-CCT_C_ as indicated by shorter neurites (Fig. 5f) and reduced complexity (Fig. 5g), consistent with CMI-dependent inhibition of neuritogenesis we observed earlier (Fig. 3g). In contrast, NLS-tagged CCAT_C_ and CCT_C_ were constrained in the nucleus, presumably acting as neuritogenic transcription factors^19^ (Fig. 5d–g). Indeed, both peptides led to the significant promotion of neurite outgrowth, whereas the longer peptide NLS-CCAT_C_ was slightly more potent than the shorter NLS-CCT_C_ (Fig. 5f), suggesting that the second TA (transcription activation) region (roughly overlapped with DCRD) may play a major role and the first TA region (largely overlapped with PCRD) would be relatively less significant (Supplementary Fig. 1). By revisiting Fig. 3, the actual effects on neurite outgrowth have been clarified to be highly dependent on subcellular localization of DCT peptides, which could be inhibitory when present in the cytosol by attenuating Ca_V_1 activities and signals, or could be neuritogenic when localized in the nucleus as transcription factors (Fig. 5h). Furthermore, such opposing effects have been confirmed with more mature neurons (>DIV 15) of which neurite outgrowth was suppressed or facilitated by CCT_D_ explicitly tagged with NES or NLS, respectively (Supplementary Fig. 10).

Cytosolic and nuclear DCT peptides are in direct opposition to each other (inhibition versus facilitation) in regulating neuritogenesis, as the plausible reason to account for the less-pronounced overall effects observed from overexpressing CCAT_C_, CCT_C_, and CCT_D_ (Fig. 3a–c). On the one hand, nuclear DCT peptides are able to promote neuritogenesis; but on the other hand, cytosolic peptides have distinct CMI potency to induce differential levels of inhibition on neurite outgrowth. Is there any mechanism to regulate/maintain the potential balance between the opposing (cytosolic versus nuclear) effects? First, the spatial distribution was examined for DCT peptides in accordance to neurite outgrowth. As depicted by the scatter plots to correlate N/C ratio with total neurite length, the peptides CCAT_C_, CCT_C_ and CCT_D_ (compared with CCT_D__V/A) spread across the cytosol/nucleus of each neuron (Fig. 5i). In contrast, the DCRD_C_ and DCRD_D_ peptides were exclusively constrained within the cytosol thus solely functioning as inhibitors of neurite outgrowth (Supplementary Fig. 11). Roughly at the same expression levels for whole cells, these peptides exhibited different patterns of subcellular distribution, as illustrated by the dynamic range between the minimum and maximum N/C ratios for each peptide. For CCAT_C_, the rather narrow range of N/C ratio is consistent with its least localization in the nucleus. In comparison, CCT_D_ appears to spread into the nucleus with a broader range of N/C ratio. We quantified the dynamic range of peptide distribution by the standard deviation (σ) of N/C ratio, which is closely correlated with CMI potency in the order of CCAT_C_, CCT_D__V/A, CCT_C_, and CCT_D_ from weak to strong (Fig. 5j). Regarding the mutant peptide CCT_D__V/A, the single-residue V/A mutation in the DCRD domain significantly attenuated its inhibitory effects on channel gating (Supplementary Fig. 12a–c), and neurite outgrowth (Supplementary Fig. 12d–f). Notably, nuclear CCT_D__V/A still promoted neurite outgrowth, similar to WT CCT_D_ in the nucleus. In agreement with the proposed σ*-CMI* correlation, CCT_D__V/A is less distributed in the nucleus compared with WT CCT_D_ (Fig. 5j); also, the clear differences between CCT_D__V/A and WT CCT_D_ resemble CCAT_C_ versus CCT_C_. One potential explanation could be that the distributions and effects of DCT peptides are subject to certain autonomous regulations in neurons, presumably by way of Ca_V_1/Ca^2+^ influx and Ca^2+^-sensitive NRD (the nucleus retention domain contained in the long or medium peptides but not in the short peptides, Supplementary Fig. 1).

### Downregulations of neuritogenesis signaling by Ca_V_1.3-encoded peptides

**Fig. 6:**
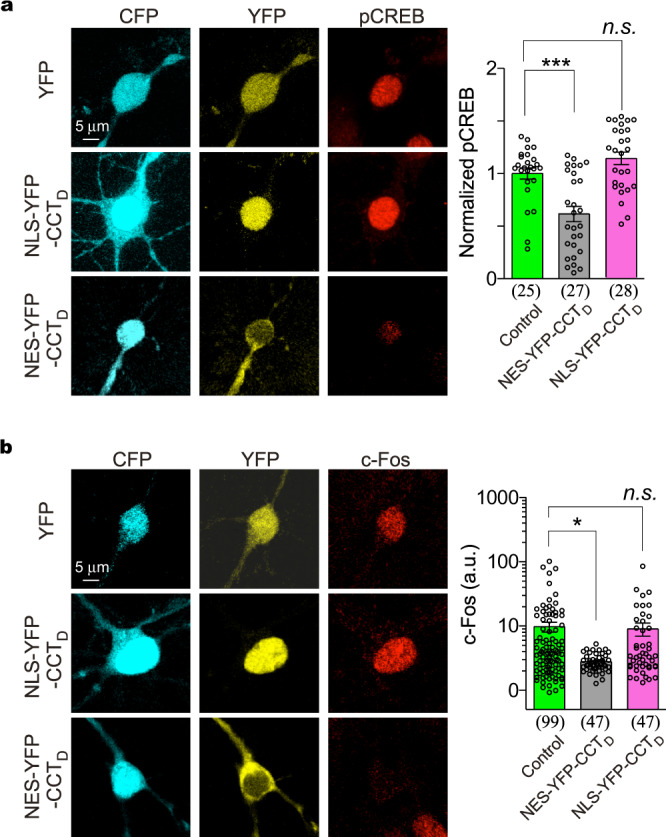
Effects of Ca_V_1.3-encoded peptides on neuritogenesis signaling in cortical neurons. **a**, **b** Inhibitory effects on pCREB and c-Fos by cytosolic peptides of exogenous CCT_D_. Confocal fluorescence images of cortical neurons expressing YFP, NLS-YFP-CCT_D_ or NES-YFP-CCT_D_. YFP indicates CCT_D_ distribution, and CFP illustrates cell bodies, respectively. Normalized pCREB (**a**) and c-Fos fluorescence (**b**) in the nuclei are summarized and compared. One-way ANOVA followed by Dunnett for post hoc test (**a**), and Kruskal-Wallis and Dunn’s non-parametric test (**b**, non-normal distribution, checked by D’Agostino & Pearson omnibus normality test) (*p < 0.05; ***p < 0.001; *n.s*. denotes not significant, *p* > 0.05). Values are represented as mean ± SEM.

The localization-dependent regulation of neuritogenesis reconciles the opposing roles of exogenous DCT peptides in neurons. Before we proceeded further with its potential Ca^2+^/Ca_V_1 dependence, we examined the key signals that are involved. For Ca_V_1.3-encoded peptides, CCT_D_ should serve as one representative form, considering that all the key domains such as NRD are included and there is no issue of weak PCRD as in CCAT_C_ (Supplementary Fig. 1). As expected, the exogenous peptides of NES-CCT_D_ demonstrated that the cytosolic peptides suppressed pCREB in direct contrast to the nuclear peptides of NLS-CCT_D_ (Fig. 6a). c-Fos, a hallmark gene of DCT effects (Fig. 1d), was also examined here for its expression driven by Ca_V_1/pCREB. Resembling pCREB, c-Fos expression was significantly reduced by NES-tagged but not by NLS-tagged peptides (Fig. 6b), consistent with that nuclear DCT peptides are able to directly serve as transcription factors to promote expression of neuritogenic genes. Next, we attempted to explore whether Ca_V_1.3-encoded peptides would play an endogenous role in cortical neurons. By anti-CT_D_ immunostaining, the subcellular distribution of CT_D_ peptides exhibited a potential correlation with neurite outgrowth (Supplementary Fig. 13a, b). In agreement with exogeneous peptide effects, endogenous CT_D_ in the cytosol appeared to suppress pCREB signaling (Supplementary Fig. 13c). The antibodies of anti-CT_D_ and anti-CT_C_ resulted into western-blot bands of differential sizes (Supplementary Fig. 14a, b and Supplementary Fig. 17a, b), supporting antibody specificity with no cross-reactivity (Supplementary Figs. 14c, 17c). Meanwhile, a bicistronic mechanism^19,43^ may underlie the peptide production from the full-length α_1CL_ or α_1DL_ transfected into HEK cells (Supplementary Figs. 14d–f, Fig. 17d–f).

In summary, Ca_V_1.3-encoded DCT peptides, if exogenously expressed in the cytosol, suppressed Ca_V_1/CREB-mediated neuritogenesis, which may represent the role of endogenous DCT peptides in cortical neurons awaiting future investigations.

### Ca_V_1/Ca^2+^ influx and CMI are critical to peptide distributions in neurons

**Fig. 7:**
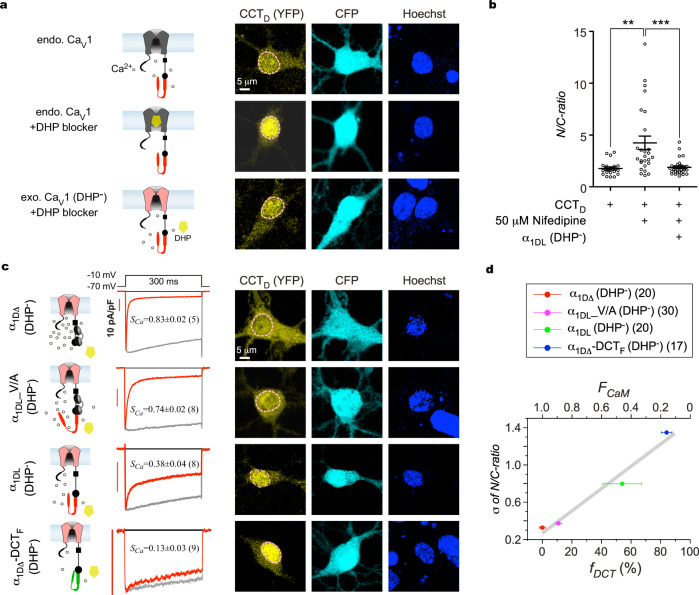
Unique importance of Ca_V_1 to subcellular DCT distributions in neurons unveiled by CMI. **a**, **b** Effects of dihydropyridine (DHP). As shown in the cartoon (**a**, left), endogenous Ca_V_1 channels in cortical neurons mediate Ca^2+^ influx (upper); DHP (50 μM nifedipine) specifically blocked cortical Ca_V_1 channels thus reducing Ca^2+^ influx (middle); and overexpression of α_1DL_ (DHP^-^) channels (mutant Ca_V_1.3 insensitive to DHP) rescued Ca^2+^ influx in the presence of 50 μM nifedipine (bottom). Exemplary fluorescence confocal images through three channels: YFP (CCT_D_ distribution), CFP (cell contour) and Hoechst (nuclear envelop) (**a**, right). N/C ratios of individual neurons from the three groups: control, DHP, and α_1DL_ (DHP^-^) with DHP (**b**). **c** Ca_V_1 variants containing DCT of different potency regulated cytosolic-nuclear distribution of CCT_D_. In addition to CCT_D_, cortical neurons were overexpressed with one of the four DHP-resistant Ca_V_1.3 variants: α_1DΔ_ (lacking DCT), α_1DL__V/A (with the key mutation of Valine to Alanine at DCRD), α_1DL_ (control), and α_1DΔ_-DCT_F_ (chimera with ultra-strong DCT). These Ca_V_1.3 variants were characterized by exemplar current traces from HEK293 cells expressing the variants illustrated in the cartoons. Cortical neurons were treated with 50 μM nifedipine to block endogenous Ca_V_1 channels while sparing the exogenous DHP^-^ Ca_V_1.3 channels. Confocal images depict cytosolic-nuclear localizations of CCT_D_ peptides in a similar fashion to (**a**). **d** Correlation between DCT inhibition of each Ca_V_1.3 variant and spatial dynamics for CCT_D_ peptides in cortical neurons. DCT/CMI potency (DCT-bound fraction *f*_*DCT*_) here is directly linked to inactivation (fraction of apoCaM-bound channels, *F*_*CaM*_): *f*_*DCT*_ = 1 − *F*_*CaM*_ (Eq. E5 in Methods). DCT/CMI potency and standard deviation of CCT_D_ distribution (σ) exhibited a tight correlation (linear fit, *R*^*2*^ = 0.95). One-way ANOVA followed by Bonferroni for post hoc tests were used for (**b**) (**p < 0.01; ***p < 0.001). Data are represented as mean ± SEM.

We discovered that subcellular distributions of DCT peptides are subject to autoregulation in accordance with peptide *CMI*, potentially through DCT inhibition of Ca_V_1/Ca^2+^ influx (Fig. 5i, j). In support, nuclear export of DCT peptides is enhanced by intracellular Ca^2+^ rise as previously reported^19,40^. Ca^2+^ influx, via Ca_V_1 channels in particular, is critical to subcellular distributions (or cytosol-nucleus translocation) of DCT peptides, which was confirmed in this work (Supplementary Fig. 15). When Ca_V_1 activities under basal conditions (5 mM [K^+^]_o_) were blocked by 50 μM nifedipine (DHP derivative), the tendency of CCT_D_ to translocate into the nucleus was significantly enhanced. In addition, N/C ratio of CCT_D_ returned back to the control level when DHP-insensitive channels (α_1DL_ DHP^-^) were employed instead (Fig. 7a, b), supporting the unique importance of Ca_V_1 in cytosol-nucleus translocation of CCT_D_. In cortical neurons (supplied with 50 μM nifedipine) where endogenous Ca_V_1 channels were replaced with DHP-insensitive channels, we further investigated whether different DCT motifs contained within the channels (through differential overall *CMI*) could produce any effect on peptide translocation. Four DHP-resistant Ca_V_1.3 variants, in the order of α_1DΔ_, α_1DL__V/A, α_1DL_ and α_1DΔ_-DCT_F_, exhibited increasingly stronger DCT effects, i.e., leading to weaker activation/inactivation, and less fraction of apoCaM-bound channels (Fig. 7c). In this context, covalently-linked (intramolecular) DCT motifs comply with the same principle as standalone (intermolecular) DCT peptides to induce inhibitory effects (CMI), both able to elevate DCT-bound fraction (and reduce apoCaM-bound fraction) of channels, thus inhibiting Ca^2+^ influx and Ca^2+^/Ca_V_1 signaling. Since α_1DΔ_-DCT_F_ (DHP^-^) channels would have the largest DCT-bound fraction (*f*_*DCT*_) when expressing in neurons, more CCT_D_ peptides translocated into the nucleus and exhibited the largest variation in peptide’s localization (σ of N/C ratio) (Fig. 7d). In contrast, for neurons expressing the α_1DΔ_ DHP^-^ channels (the weakest *CMI* due to completely lacking DCT), the least nuclear retention of CCT_D_ (the smallest σ) was observed. This is presumably due to the fact that Ca^2+^ influx via Ca_V_1 channels and the related Ca^2+^-sensitive nuclear export of CCT_D_ would be the most pronounced for α_1DΔ_ DHP^-^ among the four variants.

Effective CMI (incorporating both intra- and inter-molecular CMI) could be tuned by a series of standalone DCT peptides (acting on the same channel Ca_V_1.3, Fig. 5j), or by replacing the full-length Ca_V_1.3 with various DCT motifs (modulated by the same peptide CCT_D_, Fig. 7d). Taken together, a tight correlation has been unveiled between DCT-peptide localization and Ca_V_1-channel activities, where the central factor is overall DCT inhibition of Ca_V_1 (effective CMI). To this point, Ca_V_1-encoded DCT peptides comply with a type of self-regulatory scheme that DCT distributions are adjusted between the cytosol versus the nucleus through DCT inhibition of Ca_V_1 channels.

## Discussion

**Fig. 8:**
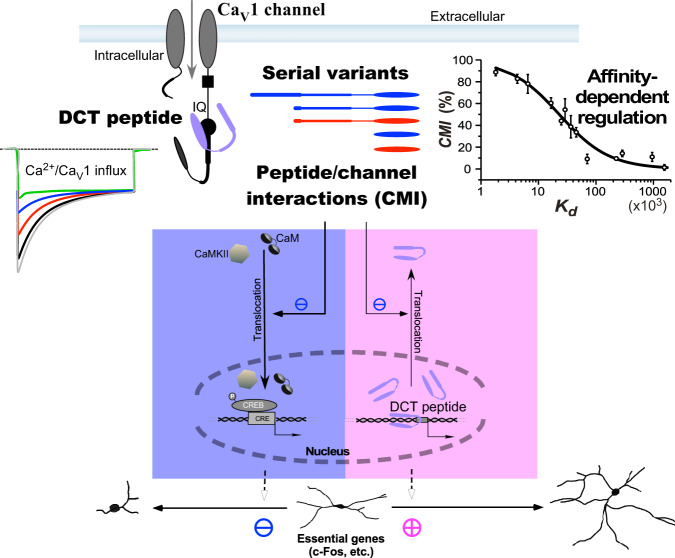
Cytosolic DCT peptides regulate Ca_V_1 channel- and nuclear DCT-mediated neuritogenesis. Regardless of the distinctions in mechanisms of production, molecular compositions, or modulatory effects, diverse peptides have been unified into a tuning curve of the central principle: DCT/Ca_V_1 affinity-dependent inhibition of the channel activity-neuritogenesis coupling, which has been demonstrated by representative Ca_V_1.3 channels in cortical neurons. A series of DCT peptide variants (color-coded to illustrate the difference in origin), by interacting with Ca_V_1 channels, inhibit Ca^2+^ influx (color-coded to illustrate the difference in potency) and regulate Ca_V_1 signaling to the nucleus and gene expression, all quantitatively in accordance with peptide/channel affinity (*CMI-K*_*d*_ relationship). For Ca_V_1 channel bound with CaM at the preIQ-IQ domain, cytosolic DCT peptides compete against CaM to form peptide-channel complex. The capability of DCT competition is quantified as CMI potency to represent the fraction of channels being switched from CaM-bound to DCT-bound. DCT-channel affinities or CMI potencies are drastically different among serial peptide variants including those endogenous to native cells, varying in size (long, medium or short) and/or origin (Ca_V_1.1-1.4). In close correlation with CMI potency, DCT peptides inhibit Ca_V_1 gating and Ca^2+^ influx, reduce nuclear translocation of key signaling molecules CaM (and CaMKII), and attenuate Ca_V_1-mediated transcription (e.g., pCREB) and expression of essential genes (e.g., c-Fos), eventually leading to inhibition of neuritogenesis (in the blue shade). As collateral effects (in the pink shade), CMI also downregulates nuclear export of DCT peptides, and thus facilitates gene transcription directly mediated by nuclear DCT peptides, leading to promotion of neurite arborization and extension. In summary, we discover cytosolic DCT inhibition of the Ca_V_1 activity-neuritogenesis coupling, which is in direct opposition to neurotrophic signaling of nuclear DCT.

In this study, we systematically examined a series of DCT-encoded peptides across the Ca_V_1 family; and by focusing on the representative Ca_V_1.3 in cortical neurons, we unveiled that DCT peptides through CMI inhibit the signaling cascade from Ca_V_1 to neuritogenesis. One determining factor of the overall CMI effects is the DCT affinity with Ca_V_1, contributed by both PCRD and DCRD segments of the peptide-channel complex. In parallel, the reduction of Ca^2+^ influx by cytosolic DCT is in favor of nuclear localization of DCT acting as neurotrophic transcription factors. In all, Ca_V_1 channel activities, Ca_V_1 channel-mediated signaling to the nucleus, gene transcription related to neuritogenesis, and Ca_V_1/Ca^2+^-sensitive nuclear export of DCT are all downregulated by cytosolic DCT peptides that bind and inhibit Ca_V_1 channels (Fig. 8).

In this study, our major strategy was to utilize a series of representative DCT peptides covering the major variants across the Ca_V_1 family. The central hypothesis has been that the DCT peptides with apparent distinctions share the same principles: the capabilities to downregulate Ca_V_1 activity-dependent neuritogenesis via the interactions between DCT peptides and Ca_V_1 channels. On the other hand, it would be unrealistic to exclusively examine all the signals or events along the whole pathway. Instead of multiple checkpoints with less rigor, our resolution was to focus on the key signals or indices, e.g., Ca_V_1 gating or neurite morphology, but taking advantage of multiple peptides with a gradient of binding affinities and neuronal effects for quantitative consolidations.

Although some Ca_V_1 inhibitors such as dihydropyridine (DHP) do exert inhibitory effects on Ca_V_1-dependent signaling and neuritogenesis^10,11^, it is still a difficult task to identify effective Ca_V_1 inhibitors for Ca_V_1-dependent neuritogenesis. First, the linkage from Ca^2+^ influx to downstream signaling is not guaranteed, i.e., the potential decoupling between Ca_V_1 channels and gene expressions, known as flux independence. In fact, the channel pore blocker Cd^2+^ and the gating blocker nimodipine may behave very differently in their effects on pCREB signals^59^. Also, instead of neuritogenic effects, Ca_V_1 agonist Bay-K-8644 causes neural toxicity^60,61^. In Timothy syndrome, gain-of-function Ca_V_1.2 mutations that promote Ca^2+^ influx cause neural damages due to ectopic activation of retractive signals^17^. Therefore, careful experiments and analyses are required to link modulation of Ca_V_1 to neuritogenesis. Second, multiple factors besides Ca^2+^ influx should work synergistically to ensure the complete signaling cascade. Less noticeable factors, e.g., voltage-dependent conformational changes of α or β channel subunits, may also play important roles to ensure proper signaling from Ca_V_1 to the nucleus^59,62^. Channel inhibitors which only reduce the Ca^2+^ influx may not attenuate neuritogenesis as effectively as expected. In this context, we have unveiled a class of Ca_V_1-encoded peptide inhibitors endogenously present in neurons that effectively and consistently downregulate Ca_V_1-dependent neuritogenesis, presumably by stripping apoCaM from the IQ domain of the channel.

The effects of DCT on channel gating appear to be divergent among Ca_V_1 family members before this work. Whether CCTs could affect Ca_V_1.1’s functions has been debated, perhaps due to different cellular environments and/or different truncation sites in these studies^26,57^. Moreover, for Ca_V_1.2 channels, it has been reported that DCT_C_ attenuates channel activation but does not affect Ca^2+^-dependent inactivation^22^, inconsistent with attenuation of inactivation evidenced from other reports^24,27^. In contrast, DCT_D_ causes strong attenuation concurrently on both activation and inactivation, as the resolution of the contradictory effects on Ca^2+^ influx; and DCT_F_ and DCT_D_ resemble each other except that DCT_F_ inhibition is of even higher potency^20,21,24^. Here, we have provided a tuning scheme of CMI unified across Ca_V_1.1-1.4 DCT (Fig. 8), demonstrated by representative Ca_V_1.3 channels, which is expandable onto Ca_V_1.2 (Supplementary Fig. 16) and other Ca_V_1 channels. In particular, DCRD_S_ and DCRD_C_ are actually able to exert inhibition (CMI) of substantial potency, as opposed to previous observations or estimations although relatively less potent than DCRD_D_ and DCRD_F_. Importantly, we have clarified that the existing discrepancy in CMI potency among the DCT variants is critically dependent on the differences in PCRD isoforms. The weak effects of DCT_S_ and DCT_C_ are mainly attributed to PCRD_S_ or PCRD_C_. Future structure-function analyses are needed to identify key PCRD residues and related mechanisms in detail. Two arginine residues reported earlier (R1696 and R1697 of Ca_V_1.2) serve as the potential candidates on PCRD^22^. A few residues away from the above sites, i.e., S1575/T1579 or S1700/T1704 on Ca_V_1.1 and Ca_V_1.2 respectively, may provide some additional clues^63,64^. Our tuning curves could make the predictions for *CMI* and/or *K*_*d*_ of diverse DCT peptides in principle (Fig. 4g and Supplementary Fig. 7). For example, regarding CCT_S_ peptides generated by cleavage of Ca_V_1.1 in skeletal muscle^36^, its CMI potency by estimations would be moderate if acting on Ca_V_1.3 (PCRD_D_), and weak on Ca_V_1.1 (PCRD_S_) according to *CMI-K*_*d*_ relationship. Hence, it is unlikely that (cytosolic) CCT_S_ could cause any strong inhibition of Ca^2+^ influx via Ca_V_1.1 in smooth muscle cells, which may help elucidate the existing arguments^36,57^. Despite important progress in Ca_V_1 structures, none of these structures has acquired atomic details on DCT^52–54^, which are foundational to understand DCT functions^20,24^. Our data here provide both the properties in common and the critical differences among DCT variants. FRET binding and electrophysiology data suggest that DCT is subject to the tight competition with CaM before the channel permits Ca^2+^, based on which we postulate that DCT peptides may acquire the (apo)CaM-like structures. In this context, DCRD and PCRD may mimic the C- and N-lobe of CaM respectively. First, apoCaM usually binds the target (such as the IQ domain of neuromodulin or neurogranin) with its C-lobe^65^; similarly, DCRD as one of the two helical subdomains plays a dominant role in the DCT/apoCaM competition (for binding the IQ domain of Ca_V_1). In comparison, PCRD appears to be assistive, e.g., to properly anchor DCRD in the close vicinity (of the channel). Second, the interactions between the CaM-binding motif and EF-hand containing CaM-like proteins are mainly mediated by charged and aromatic/hydrophobic residues^65,66^, which are also similarly enriched in DCT. The functional and structural details of CMI/DCT would advance our understanding about how apoCaM binds Ca_V_1 and promotes its functions^23,67^.

In this work, a neurotrophic role has been confirmed for DCT peptides localized in the nucleus; meanwhile, cytosolic DCT peptides inhibit neurite outgrowth. Potentially, the overall effects may constitute a homeostatic balance sustained by two signaling opponents (cytosolic versus nuclear) in neurite morphogenesis (Fig. 3b, Fig. 5h and Supplementary Fig. 13b). DCT/CMI inhibits the Ca_V_1 activity-neuritogenesis coupling represented by the following crucial signals or events: translocation of CaM/CaMKII from cytosol to nucleus, phosphorylation of CREB, and transcription and expression of hallmark genes (e.g., c-Fos)^11,14,49,68,69^. Higher CMI potency leads to less Ca^2+^ influx via Ca_V_1, eventually causing more pronounced retraction of neurites. On the other hand, less Ca^2+^ influx resulted from potent DCT peptides tends to cause more nuclear retention due to Ca^2+^-dependent nuclear export of DCT peptides. Nuclear DCT peptides as transcription factors drive the expression of a spectrum of neurotrophic genes^19^. Notably, DCT in the nucleus is autonomously regulated by DCT in the cytosol through its inhibition on Ca_V_1. For example, under our experimental conditions of basal (channel/neuron) activities, CCAT_C_ exhibits ultraweak inhibition of Ca_V_1 but with a larger fraction of cytosolic distribution, opposed to nuclear DCT peptides of a relatively smaller fraction. CCT_D_ is much more potent in Ca_V_1 inhibition but a relatively small cytosolic fraction (in opposition to nuclear CCT_D_ of a larger fraction), hence the tendencies of retraction/maintenance versus outgrowth could be substantially balanced out. For CCAT_C_ or CCT_D_, similar autoregulatory mechanisms may account for the rather mild CMI effects on neuritogenesis (Fig. 3b). We postulate that CMI regulation of endogenous DCT distribution would be employed to maintain a delicate balance for neuritogenesis, the setting points of which may vary with developmental stages, external stimuli or cues, and actual peptide variants, in addition to expression levels. Such tuning scheme of homeostasis is expected to generally apply to other types of neurons. Besides Ca_V_1.3 and Ca_V_1.3-encoded DCT peptides (e.g., CCT_D_ and CT_D_) as the focus of this work, Ca_V_1.2 and Ca_V_1.2 DCT peptides (e.g., CCAT_C_ and CCT_C_) are widely expressed in the brain^3,43,44^. Compared with Ca_V_1.3, Ca_V_1.2 channels should have less potent CMI effects due to the weak PCRD_C_ motif, as another reason for this work to focus on Ca_V_1.3. In cerebellar granule cells, CCAT_C_ peptides serve as nuclear transcription factors that promote neurite ourgrowth^19^. CCAT_C_ has also been evidenced in cell nuclei of the cerebellum and thalamus in embryonic brain, exporting to the cytosol along with aging and development^41^. CCT_C_ by proteolysis has been found in hippocampus neurons^37^, and hippocampal Ca_V_1 channels are required for normal neurogenesis^45^.

In addition to Ca_V_1, Ca_V_2.1 and Ca_V_3.2 could encode peptides targeting the nucleus to regulate gene transcription by the bicistronic mechanism, which might be conserved across the superfamily of voltage-gated Ca^2+^ channels^41–43,70^. C-terminal fragments of α_1A_ act as transcription factors to promote neuronal development^42,43^, resembling the effects of Ca_V_1 DCT in the nucleus. Unlike the dual roles of Ca_V_1 DCT peptides in this study, Ca_V_2 CT has not been found to have any effect on channel gating^20,71^. Besides the autoregulatory scheme proposed here (Fig. 8), some other forms of feedbacks may also exist, e.g., Ca_V_1.2-encoded peptides could reduce the transcription of Ca_V_1.2 gene when located in the nucleus of cardiac myocytes^38,72^. Although no such downregulation has been evidenced from Ca_V_1.3 in either recombinant systems or cortical neurons, more direct examinations are necessary to confirm the actual expression of functional Ca_V_1.3 channels in different scenarios. Ca_V_1-encoded polypeptides exhibit Ca^2+^-dependent nuclear export, with the aid of its nucleus retention domain NRD^19^, also supported by the distinctions between the short and long/medium peptides demonstrated in this study (Fig. 5h, i and Supplementary Fig. 11). Ca^2+^-dependent DCT translocation is particularly sensitive to Ca^2+^ via Ca_V_1 (Fig. 7), while the exact mechanisms of DCT translocation are still awaiting future investigations^19^.

The specificity of the antibodies is critical given that non-specific activities of Cav antibodies are not uncommon, in part due to the low expression levels of these membrane channels compared to other proteins. In the future, rigorous validations, e.g., with Ca_V_1.3 or Ca_V_1.2 knock-out (ideally conditional knock-out) neurons and/or the control of blocking peptides, are expected to confirm the western-blot and immunostaining data. A set of consistent data would strengthen the conclusions, if multiple approaches including electrophysiology, biochemistry and imaging could be combined together. For future work, additional methods/tools are expected, such as Ca_V_1.3 antibodies with knock-out validations. Meanwhile, due to the compensatory effects on Ca^2+^ channels in Ca_V_1.3^-/-^ and Ca_V_1.2^-/-^ mice as reported^73,74^, cautions also need to be taken in interpreting the data from these knock-out mice. Alternatively, knock-in mice such as Ca_V_1.2 DHP^-/-^ and/or Ca_V_1.3 DHP^-/-^ may be advantageous for the purpose of identifying and isolating Ca_V_1.3 and Ca_V_1.2 channels.

Our data demonstrate that inhibition of Ca_V_1/Ca^2+^ influx is highly correlated with attenuation of Ca_V_1 signaling and neuritogenesis. As mentioned earlier, for particular modulation or perturbation of Ca_V_1 channels, it may not be as effective as expected for downstream signals and neuritogenesis. In this work, based on the fact that apoCaM/Ca_V_1 binding is the critical linkage from channel gating to nuclear signaling^20,23,75^, we propose DCT/CMI as an outstanding modality with high specificity and effectiveness compared to other Ca_V_1 inhibitors known thus far. Particularly targeting Ca_V_1, DCT consistently generates inhibitory effects across the signaling cascade. We expect that small molecules or biologics mimicking DCT/CMI would provide new interventions for potential therapeutics of diseases related to the Ca_V_1 activity-neuritogenesis coupling. Both Ca_V_1 channels and neurite outgrowth are involved in a variety of neuropsychiatric and neurodegenerative diseases, such as autism, bipolar disorder, schizophrenia, Parkinson’s disease, and Alzheimer’s disease^76–78^. Ca^2+^ dysregulations associated with Ca_V_ have gained increasing support for its close relevance to neurodegenerative diseases, known as the ‘Ca^2+^ hypothesis’^79,80^. However, DCT peptides in these diseases are largely unexplored despite the observations indicating that the amount and distribution of DCT peptides are age-dependent^19,41^. In this regard, there are still unresolved questions pertaining to Ca_V_1 channels, DCT peptides, and Ca_V_1/DCT-dependent neuritogenesis. Exemplars of such questions include: whether and why Ca_V_1 genes (compared with other Ca_V_) play uniquely important roles in certain pathological processes; whether and how DCT and CMI (compared with other modulations) would play unique roles; whether DCT is prone to disease-associated mutations and how exactly mutant DCT would affect healthy neurons; and eventually what need to do to rescue the defective DCT and neurons. Notably, the expression levels of CaM are downregulated in Parkinson’s disease and Alzheimer’s disease^77,81^, for which the overall inhibitory effects of DCT peptides on Ca_V_1 and Ca_V_1-mediated downstream signals would be even more profound due to less apoCaM competition (Fig. 8).

## Methods

### Molecular biology

The plasmids of channels and peptides were constructed from α_1S_ (rabbit Ca_V_1.1, NM_001101720.1, Genbank^TM^ accession number), α_1C_ (human Ca_V_1.2, AF465484.1), α_1DL_ (human Ca_V_1.3 α_1DL__human, EU363339.1; or rat Ca_V_1.3 chimera α_1DL__rat: backbone a.a. 1-1625 from rat AF370009.1 and DCT_D_ 1626-2155 from a.a. 1674-2203 of rat NM_017298.1), and α_1F_ (human Ca_V_1.4 NP005174). In particular, α_1DΔ_ was generated by truncation of α_1DL_ (rat AF370009.1) with a unique XbaI site following the IQ domain (ending with G1625). For chimeric α_1DΔ_-PCRD_D_ and α_1DΔ_-DCT_F_, desired segments (PCRD_D_ from 1626-1780 in α_1DL__rat and DCT_F_ from 1596-1966 in α_1F_) were PCR-amplified with SpeI and XbaI sites and cloned into aforementioned α_1DΔ_. Rat Ca_V_1.3 DHP^–^ was generated by single point mutation T1033Y^46^ on α_1DΔ_, α_1DL__rat, α_1DL__V/A (V2075A in α_1DL__rat) or α_1DΔ_-DCT_F_, respectively. α_1DL_-Flag was generated by fusing PCR-amplified 3xFlag (DYKDHDGDYKDHDIDYKDDDDK) to the C-terminus of α_1DL__rat by KpnI and SacII. α_1CL_-Flag was generated by replacing the EGFP in a customized pEGFP-N1 vector (modified by inserting a 3xHA tag before MCS) with PCR-amplified α_1CL__human fused with 3xFlag via XhoI and NotI sites.

CFP/YFP-DCRD_F_ in pcDNA3 were constructed as the templates for peptide plasmids. In brief, CFP or YFP was inserted into pcDNA3 vector with the unique KpnI and NotI sites, then DCRD_F_ was fused to the C-terminus of CFP/YFP by unique NotI and XbaI sites. Other CFP/YFP-tagged constructs were generated by replacing DCRD_F_ with appropriate PCR amplified segments, via unique NotI and XbaI sites. The constructs we have made include: YFP-DCRD_F_ truncations, CFP-DCRD_S/C/D_ (DCRD_D_ from α_1DL_ human EU363339.1), YFP-preIQ_3_-IQ_D_-PCRD_S/C/D/F_ (preIQ_3_-IQ_D_ from 1576-1625 and PCRD_D_ from 1626-1780 in α_1DL__rat), CFP/YFP-PCRD_S/C/D/F_-DCRD_F_ (PCRD_D_ from 1626-1780 in α_1DL__rat), YFP-PCRD_C_-DCRD_C_ and YFP-CCAT_C_. DCRD_S/C/D_-YFP (DCRD_D_ from α_1DL__human), CCT_C_-YFP and CCT_D_-YFP (CCT_D_ from α_1DL__human) were based on another pcDNA3/YFP vector with the cloning sites of KpnI and NotI on 5’. The pcDNA3/YFP vector was made by inserting YFP into pcDNA3 vector with the unique NotI and XbaI sites. Single point mutants such as CFP/YFP-DCRD_F__V/A and CCT_D__V/A-YFP were made by overlap PCR. For DCT peptides to target nucleus or cytosol, nuclear localization signal (NLS) (PKKKRKV) or nuclear export signal (NES) (LALKLAGLDIGS) was fused to N-terminus of YFP-DCT peptides by overlap PCR, to achieve NLS-YFP-CCAT_C_, NES-YFP-CCAT_C_, NLS-YFP-CCT_C_, NES-YFP-CCT_C_, NLS-YFP-CCT_D_ and NES-YFP-CCT_D_. For 3xFlag-DCT_C/D_, 3xFlag tag was firstly inserted into pcDNA3 vector with the unique KpnI and NotI sites, then DCT_C/D_ (from α_1CL_human_ or α_1DL_human_) peptides were PCR-amplified with NotI and XbaI sites and fused to the C-terminus of 3xFlag.

### Dissection and culturing of cortical neurons

Cortical neurons were dissected from postnatal day 0 (P0, either sex) newborn ICR mice. Isolated cortex tissues were digested with 0.25% trypsin for 15 min at 37 °C, followed by terminating the enzymatic reaction by DMEM supplemented with 10% FBS. The suspension of cells was sieved through a filter then centrifuged at 1000 rpm for 5 minutes. The cell pellet was resuspended in DMEM supplemented with 10% FBS and then plated on poly-D-lysine-coated 35-mm No. 0 confocal dishes (In Vitro Scientific) or poly-D-lysine-coated coverslips. After 4 hours, neurons were maintained in Neurobasal medium supplemented with 2% B27, 1% glutaMAX-I (growth medium). Temperature of 37 °C with 5% CO_2_ was controlled in the incubator. All animals were obtained from the laboratory animal research centers at Tsinghua University and Peking University. Procedures involving animals have been approved by local institutional ethical committees of Tsinghua University and Beihang University.

### Transfection of cDNA constructs in cell lines and cultured neurons

For electrophysiological recording, HEK293 cells (ATCC), checked by PCR with primers 5′-GGCGAATGGGTGAGTAACACG-3′ and 5′-CGGATAACGCTTGCGACCTATG-3′ to ensure free of mycoplasma contamination, were cultured in 60-mm dishes, and recombinant channels were transiently transfected according to established calcium phosphate protocol^20,24^. 5 μg of cDNA encoding channel α_1_ subunit, along with 4 μg of rat brain β_2a_ (M80545) and 4 μg of rat brain α_2_δ (NM012919.2) subunits were applied to HEK293 cells. To enhance expression, cDNA for simian virus 40 T antigen (1 μg) was also co-transfected. For each additional construct, 2 μg cDNA was added. All of the above cDNA constructs were driven by a cytomegalovirus (CMV) promoter. Cells were washed with PBS 6–8 h after transfection and maintained in supplemented DMEM, then incubated for at least 48 h in a water-saturated 5% CO_2_ incubator at 37 °C before usage.

For transfection in neurons, 2 μg of cDNA encoding the desired peptides were transiently transfected by Lipofectamine 2000 (Invitrogen) for each confocal dish with a typical protocol according to the manual. The mixture of plasmids and Lipofectamine 2000 in opti-MEM was added to the Neurobasal medium for transfection. After 2 hours, neurons were maintained in Neurobasal medium supplemented with 2% B27, 1% glutaMAX-I for 48 hours.

For 2-hybrid 3-cube FRET experiments, HEK293 cells were cultured on confocal dishes. FRET cDNA constructs of 2 μg each were transfected by Lipofectamine 2000 for 6 hours. Cells were used after 24 hours.

For western blot experiments, CHO (Cell Resource Center, IBMS, CAMS/PUMC) or HEK293 cells were cultured on 60 mm dishes. cDNA constructs were transfected by Lipofectamine for at least 6 hours. Cells were collected after 2 days.

### Whole-cell electrophysiology

Whole-cell recordings of transfected HEK293 cells were performed at room temperature (25 °C) using an Axopatch 200B amplifier (Molecular Devices). Electrodes were pulled with borosilicate glass capillaries by a programmable puller (P-1000, Sutter Instrument) and heat-polished by a microforge (MF-830, Narishige), resulting in 2–5 MΩ resistances before 70% of compensation. The internal/pipette solution contained (in mM): CsMeSO_3_, 135; CsCl, 5; MgCl_2_, 1; MgATP, 4; HEPES, 5; and EGTA, 5; with ~290 mOsm adjusted with glucose and pH 7.3 adjusted with CsOH. The extracellular/bath solution contained (in mM): TEA-MeSO_3_, 135; HEPES, 10; CaCl_2_ or BaCl_2_, 10; with ~300 mOsm, adjusted with glucose and pH 7.3 adjusted with TEA-OH, similar to the previous protocols^24^. Whole-cell currents were generated from a family of step depolarizations (−70 to +50 mV from a holding potential of −70 mV and step increase of 10 mV). Current traces were recorded at 2 kHz low-pass filtering in response to voltage steps with minimum interval of 30 s. P/8 leak subtraction was used throughout. Ca^2+^ current was normalized over different cells by cell capacitance (*C*_*m*_, in pF), and the current amplitude (peak, 50 ms or 300 ms, in pA/pF) was measured at −10mV.

Neuronal patch-clamp recording was performed according to our previous protocol^51^. In brief, isolated cortical neurons were cultured in coverslips. To record neuronal Ca_V_1.3 current, neurons were pre-incubated in Tyrode’s solution containing 1 μM nimodipine (Sigma-Aldrich), 1 μM ω-conotoxin GVIA (Sigma-Aldrich, or alomone labs) and 1 μM ω-conotoxin MVIIC (Sigma-Aldrich, or alomone labs) for 30 min to block endogenous Ca_V_1.2, N- and P/Q-type Ca^2+^ current, according to the cocktail recipes^75,82,83^. Under the conditions of our evaluations (−10 mV, full cocktail recipes), Ca_V_1.3 current appeared to be the dominant component (~80%) after the treatment, and Ca_V_2.3 (16%) and Ca_V_1.2 (4%) contributed to the rest (details see Supplementary Fig. 2). The voltage ramp protocol (holding at −60 mV, ramping from −60 to +50 mV at 0.2 mV/ms) was applied to cortical neurons in the bath solution containing 10 mM Ba^2+^. The resulted *I-V* curves were fitted by Boltzmann-based equations (OriginPro) to obtain the half-activation voltage (*V*_*half*_) of voltage-dependent channel activation. Isradipine (Sigma-Aldrich) at 100 nM was used to further isolate/confirm Ca_V_1.3 currents, and 20 μM isradipine would eliminate all the Ca_V_1 (Ca_V_1.2 and Ca_V_1.3) currents^84^. Treated neurons were recorded in various bath solutions containing appropriate blockers within one hour.

### 2-hybrid 3-cube FRET

2-hybrid 3-cube FRET experiments were carried out with standard protocols similarly shared by several groups^20,24,56^. Briefly, experiments were performed on an inverted epi-fluorescence microscope (Ti-U, Nikon), with computer-controlled filter wheels (Sutter Instrument) to coordinate with diachronic mirrors for appropriate imaging at excitation, emission, and FRET channels. The filters used in the experiments were excitation: 438/24 (FF01-438/24-25, Semrock) and 480/30 (FITC, Nikon); emission: 483/32 (FF01-483/32-25, Semrock) and 535/40 (FITC, Nikon); dichroic mirrors: 458 nm (FF458-Di02-25 × 36, Semrock) and 505 nm (FITC, Nikon). Fluorescence images were acquired by Neo sCMOS camera (Andor Technology) and analyzed with 3^3^-FRET algorithms coded in Matlab (Mathworks), mainly based on the following formula:

E1 $${FR}=1+\frac{{FR}_{{{\max }}}-1}{1+\frac{{K}_{d}}{{D}_{{free}}}}$$

*FR*_*max*_ represents the maximum FRET ratio, and *D*_*free*_ denotes the equivalent free donor (CFP-tagged) concentration. *K*_*d*_ (effective dissociation equilibrium constant) is calculated from an iterative procedure to evaluate the binding affinity for each pair of binding partners. FRET imaging experiments were performed with HEK293 cells in Tyrode’s buffer containing 2 mM Ca^2+^.

### Confocal fluorescence imaging and analysis

Cultured neurons were transfected with CFP (to label the soma area and neurites) and DCT peptides tagged with YFP on 5^th^ day (DIV-5) and used on DIV-7, or transfected on DIV 12-15 and used on DIV 15–18 (Supplementary Fig. 10). Neurons were loaded with Hoechst 33342 for 5 min to label the nuclei and then imaged by Zeiss LSM710 confocal Scanning Microscope. Fluorescent intensity was quantified and analyzed with ImageJ (NIH). Calculation of nuclear intensity was based on the nuclear contour indicated by Hoechst 33342. Cytosolic intensity was calculated by intermediate region between nucleus and plasma membrane. N/C ratio of DCT peptides was calculated by the ratio of fluorescence intensity (nuclear/cytosolic). Measurements of the total length and *Sholl* analysis for neurites were performed with Imaris 7.7.2 (Bitplane) through CFP channel. Only non-overlapping neurons were selected for analysis of morphogenesis. Neurite tracings were depicted with Imaris 7.7.2 and further processed with Photoshop 7.0 (Adobe).

To observe the cytosolic-nuclear translocation of DCT peptides, neurons were pre-incubated in 5 mM [K^+^]_o_ solution (130 mM NaCl, 5 mM KCl, 1 mM MgCl_2_, 15 mM HEPES, 2 mM CaCl_2_, at 300 mOsm adjusted with glucose) and perfused with 40 mM [K^+^]_o_ solution (95 mM NaCl, 40 mM KCl, 1 mM MgCl_2_, 15 mM HEPES, 2 mM CaCl_2_, at 300 mOsm adjusted with glucose) or 5 mM [K^+^]_o_ with 50 μM Nifedipine for 0.5-1 hour, then washed out by 5 mM [K^+^]_o_ when needed. For the experiments with DHP-insensitive variants of Ca_V_1, neurons were incubated with 50 μM Nifedipine for at least 1 hour, and neurons without clear damages were selected to calculate N/C ratio for the peptides.

Analyses on neurite morphology and cytosolic-nuclear translocation were performed over cultured neurons from at least two culture preparations and two independent experiments, adding up to the total number for each data group (20 cells or more).

### Immunocytochemistry

DIV-5 cultured cortical neurons were transfected with DCRD_F_ and used on DIV-7. Firstly, to stimulate neurons, 1 μM TTX (sodium channel blocker) was applied to neurons for 6 hours to suppress action potential. 40 mM [K^+^]_o_ solution containing 1 μM TTX was applied for 5 min for CaM staining, or 30 min for pCREB staining before fixation. To measure desired signals in physiological condition, neurons were maintained in growth medium until fixation. Secondly, neurons were rapidly rinsed with ice-cold PBS and fixed with ice-cold 4% paraformaldehyde in PBS (pH 7.4) for 15–20 min. Fixed neurons were washed by ice-cold PBS for 3 times and permeabilized with 0.3% Trition X-100 for 5 minutes. Then neurons were blocked by 10% normal goat serum in PBS for 1 hour and incubated with the primary antibodies overnight at 4 ^o^C. The following antibodies were used: CaM (Rabbit mAb #5197-1, Epitomics, Species Cross-Reactivity: Human, Mouse, Rat, Dilutions: 1:500 in PBS)^51^, pCREB (Rabbit mAb #9198, Cell Signaling Technology, Species Cross-Reactivity: Human, Mouse, Rat, Dilutions: 1:500 in PBS)^49,51^, c-Fos (Rabbit mAb [EPR21930-238] #ab222699, Abcam, Species Cross-Reactivity: Mouse, Human, Dilutions: 1:1000 in PBS, https://www.abcam.com/nav/primary-antibodies/rabbit-monoclonal-antibodies/c-fos-antibody-epr21930-238-ab222699.html), and Ca_V_1.3 CT (a.a. 2025-2161) (Mouse mAb [N38/8] #ab84811, Abcam, Species Cross-Reactivity: Mouse, Rat, Rabbit, Human, Dilution: 1:500 in PBS)^85^. Finally, the next day, neurons were washed with PBS for 3 times and incubated with the secondary antibodies (Goat anti-Rabbit Alexa Fluor 647, Invitrogen, Dilutions: 1:800 in PBS; Goat anti-Mouse Alexa Fluor 568, Invitrogen, Dilutions: 1:800 in PBS; Goat anti-Mouse IgG(H+L)-DyLight 488, Gene-Protein Link, Dilutions: 1:500) for 2 hours. Then neurons were washed with PBS for 3 times and treated with Hoechst 33342 (Invitrogen) for 5 min for nuclear counterstain. Mounted neurons on confocal dishes were imaged with a confocal microscope (LSM710, Carl Zeiss) and ZEN software. Nuclear and cytosolic fluorescence intensities of endogenous CaM in cortical neurons were analyzed by ImageJ (NIH). Neuronal culture preparations of each round supplied one or two independent experiments. Neurons (>15) were assayed or evaluated from at least two culture preparations and two independent experiments unless otherwise noted.

### Western blot

Cortical neurons for western blot were isolated from newborn ICR mice or from cultured cortical neurons around DIV-14. Liver tissues were from newborn ICR mice. HEK293 Cells and CHO (Chinese Hamster Ovary) cells served as the blank control or the recombinant system to express proteins. Cells lysates were prepared by lysis buffer RIPA (with protease inhibitor cocktail, cat#P1265, Applygen Tech) and centrifuged at 15,000 × g. Proteins were separated using 8% sodium dodecyl sulphate polyacrylamide gel electrophoresis and transferred to a nitrocellulose membrane for 100 min. Then nitrocellulose membrane was blocked in 5% non-fat dry milk and incubated with primary antibodies: anti-Ca_V_1.3 CT, identical to the antibody used in immunocytochemistry, Dilutions: 1:1000; anti-Ca_V_1.2 CT (a.a. 1835-2135) (Rabbit pAb #21774-1-AP, proteintech, Species Cross-Reactivity: human, mouse, rat, Dilutions: 1:1000, https://www.ptgcn.com/products/L-VOCC-Antibody-21774-1-AP.htm); anti-Ca_V_1.2 II-III loop (#ACC-003, allomone, Source: Rabbit, Species Cross-Reactivity: Human, Mouse, Rat, Dilutions: 1:1000, https://www.alomone.com/p/anti-cav1-2-antibody/ACC-003), anti-Flag (#20543-1-AP, proteintech, Source: Rabbit, Species Cross-Reactivity: recombinant protein with Flag tag, Dilutions: 1:3000); and anti-GAPDH (#P01L081, Gene-Protein Link, Source: Rabbit, Species Cross-Reactivity: Human, Mouse, Rat, Bovine, Pig, Chicken, Zebrafish, Green Monkey, Dilutions: 1:5000) overnight at 4 °C. Next, the nitrocellulose membrane was washed three times with TBST and incubated with secondary antibody (Goat anti-Mouse, # SA00001-1, Proteintech, Dilution: 1:3000; Goat anti-Rabbit, #ZRA03, YTHXbio, Dilution: 1:5000) for 1–2 h and then washed with TBST for three times again. The membrane was coved with ECL chemiluminescent liquid (#P1010, Applygen Tech) before detection with an enhanced chemiluminescence system. Three or more independent replicates were performed for each experiment.

### Definition of CMI potency and related curve fitting

For α_1DL_ channels with covalently-linked DCT (Figs. 2,  7), there are two subgroups: DCT-bound and apoCaM-bound. For these channels subject to modulation by standalone DCT peptides, CMI potency (*CMI*, in percentage) is quantified as the normalized fractional change of apoCaM-bound channels switching to peptide-bound channels (*f*_*Peptide*_):

E2 $${CMI}={f}_{{Peptide}}=\frac{\varDelta {F}_{{CaM}}}{{F}_{{CaM}}}$$

Since apo-CaM bound fraction (*F*_*CaM*_) is proportional to Ca^2+^-dependent inactivation (*S*_*Ca*_)^20^, we have:

E3 $${CMI}=\frac{{S}_{{Ca},{Control}}-{S}_{{Ca},{Peptide}}}{{S}_{{Ca},{Con}{trol}}}=1-\frac{1}{{S}_{{Ca},{Control}}}\cdot {S}_{{Ca},{Peptide}}$$

Hence, CMI potency is inversely and linearly correlated with actual inactivation of channels under peptide modulation. Meanwhile, interactions between peptides and channels should follow the relationship defined by the classical ligand-binding equation:

E4 $${CMI}={f}_{{Peptide}}=\frac{\left[{{{{{\rm{Peptide}}}}}}\right]}{\left[{{{{{\rm{Peptide}}}}}}\right]+{K}_{d}}$$

Here, [Peptide] is the concentration of DCT peptide, and *K*_*d*_ is the dissociation constant of peptide interactions with channels. Fluorescence intensities (a.u., arbitrary units) are used to calculate [Peptide] and *K*_*d*_. In our electrophysiology experiments, patch-clamped cells are supposed to have comparable (over)expression levels of peptides, thus Eq. E4 represents the relationship between *CMI* and *K*_*d*_ across a series of peptide variants (Fig. 4c). The concentration of DCT peptides ([Peptide]) on average was estimated to be ~23,000 (a.u.), by directly applying Eq. E4 to fit Fig. 4c. To estimate the binding affinities between YFP-preIQ_3_-IQ_D_ and CFP-PCRD_X_-DCRD_F_, we proceeded to perform FRET experiments between YFP-preIQ_3_-IQ_D_-PCRD_X_ and CFP-DCRD_F_. The reason for taking such indirect approach is in part due to severe aggregation (puncta) for YFP-preIQ_3_-IQ_D_ (but not for YFP-preIQ_3_-IQ_D_-P_X_) when overexpressed in cells. Correspondingly, *CMI* values of PCRD_X_-DCRD_F_ peptides to α_1DΔ_ were from Fig. 4e. *K*_*d*_ for IQ_D_ and P_D_-D_F_ was estimated to be 25-fold of the *K*_*d*_ value listed for IQ_D_-P_D_ and D_F_ in Fig. 4c. Similar calculations (25-fold) can be conducted to estimate *K*_*d*_ values for other peptides in Fig. 4f from Supplementary Fig. 7.

In Fig. 4g, results from Fig. 4c, f were combined and plotted together with the tuning curve (Eq. E4, ([Peptide] = 23,000). In addition, compound CMI effects for P_D_+P_F_-D_F_, P_D_+P_C_-D_F_, and P_D_+P_C_-D_C_ were also illustrated, based on *CMI* values from electrophysiology and estimated *K*_*d*_ values by interpolation.

Regarding full-length channels with covalently-linked DCT of its own (such as α_1DL_ and DCT mutants), DCT potency (of competing with apoCaM to bind the channel) can be derived from Eqs. E2 and E3 into similar formulas for the intramolecular CMI by the covalently-linked DCT (Figs. 5j,  7d). Considering α_1DΔ_ as the control (100% apoCaM-bound), (CMI) potency of linked DCT domains represents the fraction of channels bound with DCT (*f*_*DCT*_) for particular channel/DCT variant. We have

E5 $${CMI}={f}_{{DCT}}=1-{F}_{{CaM}}=1-\frac{{S}_{{Ca},{DCT}}}{{S}_{{Ca},{Control}}}$$

In principle, *CMI* refers to fractional changes of channels switched from apoCaM-bound to apoCaM-unbound (i.e., DCT-bound), which could be achieved by standalone DCT peptides and/or covalently-linked DCT domains in a similar fashion.

### Statistics and reproducibility

Data were analyzed in Matlab, OriginPro and GraphPad Prism software. Data were shown as mean ± SEM (Standard Error of the Mean). Unpaired or paired Student’s *t*-test (two-tailed with criteria of significance) was performed to compare two groups. One-way ANOVA followed by Dunnett or Bonferroni for post hoc tests were performed to compare more than two groups with or without a restrictive control group, respectively, provided the normal distribution of the data. Kruskal-Wallis and Dunn’s non-parametric test was performed if the data did not follow the normal distribution. D’Agostino & Pearson omnibus normality test was used before column analyses. Significance *p < 0.05; **p < 0.01; ***p < 0.001 and *n.s*. denotes ‘not significant’.

### Reporting summary

Further information on research design is available in the Nature Research Reporting Summary linked to this article.

## Supplementary information

Peer Review File

Supplementary Information

Description of Additional Supplementary Files

Supplementary Data 1

Reporting Summary

## Data Availability

The plasmids of pcDNA3-NLS-YFP-CCT_D_ (#184325), pcDNA3-NES-YFP-CCT_D_ (#184326), pcDNA3-NLS-YFP-CCT_C_ (#184327) and pcDNA3-NES-YFP-CCT_C_ (#184328) are available on Addgene. Source data underlying the figures are organized as Supplementary Data 1. Uncropped blots are presented in Supplementary Fig. 17. The data in details associated with the main figures have been deposited to Dryad (10.5061/dryad.cvdncjt63)^86^. Other data and information are available from the corresponding author upon reasonable request.
