# Supplementary material for: Cytosolic peptides encoding CaV1 C-termini downregulate the calcium channel activity-neuritogenesis coupling
Source: Commun Biol. 2022 May 19;5:484. doi: 10.1038/s42003-022-03438-1 (PMC9120191; doi:10.1038/s42003-022-03438-1)
Supplement: Supplementary file 2 — Supplementary Information [file 42003_2022_3438_MOESM2_ESM.pdf]

Supplementary Information

Cytosolic peptides encoding Ca_V1 C-termini downregulate the calcium channel activity-neuritogenesis coupling

by

Yaxiong Yang[#], Zhen Yu[#], Jinli Geng[#], Min Liu, Nan Liu, Ping Li, Weili Hong, Shuhua Yue, He Jiang, Haiyan Ge, Feng Qian, Wei Xiong, Ping Wang, Sen Song, Xiaomei Li[*], Yubo Fan[*] and Xiaodong Liu[*]

[#] Equal contributions

[*] Corresponding authors:

li-xiaomei@mail.tsinghua.edu.cn; yubofan@buaa.edu.cn; liu-lab@buaa.edu.cn

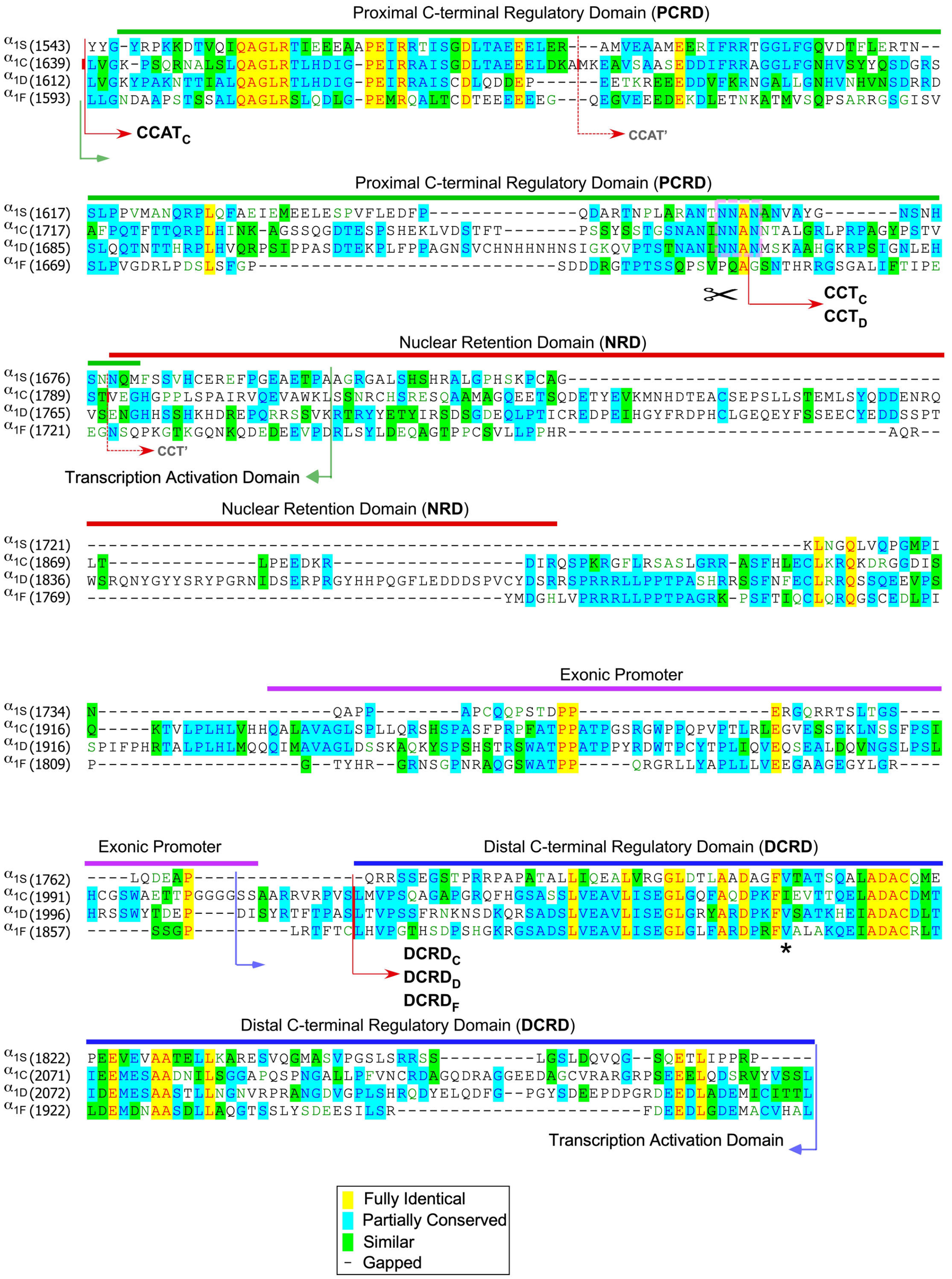

Supplementary Figure 1. Alignment of DCT variants across the Ca$_V$1 family.

The sequences of DCT were aligned for Ca$_V$1.1 (α_{1S}, NM_001101720.1), Ca$_V$1.2 (α_{1C}, AF465484.1), Ca$_V$1.3 (α_{1D}, EU363339.1) and Ca$_V$1.4 (α_{1F}, NP005174), with GenBank accession numbers in parentheses. Key domains of PCRD, NRD or DCRD are indicated with solid lines of different colors; and the representative long (~60 kDa or more) peptide CCAT$_C$[1], and medium-size (~40 kDa)

peptides CCT_C[2] and CCT_D[3], are encoded by the sequences starting from the arrows (red) to the very end (stop codon). Similarly, short (~15 kDa) peptides including $DCRD_C$, $DCRD_D$ and $DCRD_F$ in this study are also indicated by the red arrow[4]. Red arrows in dash lines are to indicate alternative versions of long and medium peptides (marked as CCAT' and CCT') documented in previous studies[2, 5], supposed to functionally resemble their extended versions $CCAT_C$ and CCT_C, respectively. Notably, PCRD is approximately overlapped with CCAT's N-terminal transcription activation domain (indicated by green arrows); and DCRD is approximately overlapped with CCAT's C-terminal transcription activation domain (indicated by blue arrows)[1]. Sequence homology from high to low is categorized as yellow (fully identical)，cyan (partially conserved), or green (similar) and – (gapped or different). Black scissor is to indicate the cleavage sites for potential proteolysis in the cell[6, 7]. Black asterisk highlights the valine residue (i.e., unveiled by Valine to Alanine or V/A mutation) critical to DCRD functions[8].

Related to Fig. 1.

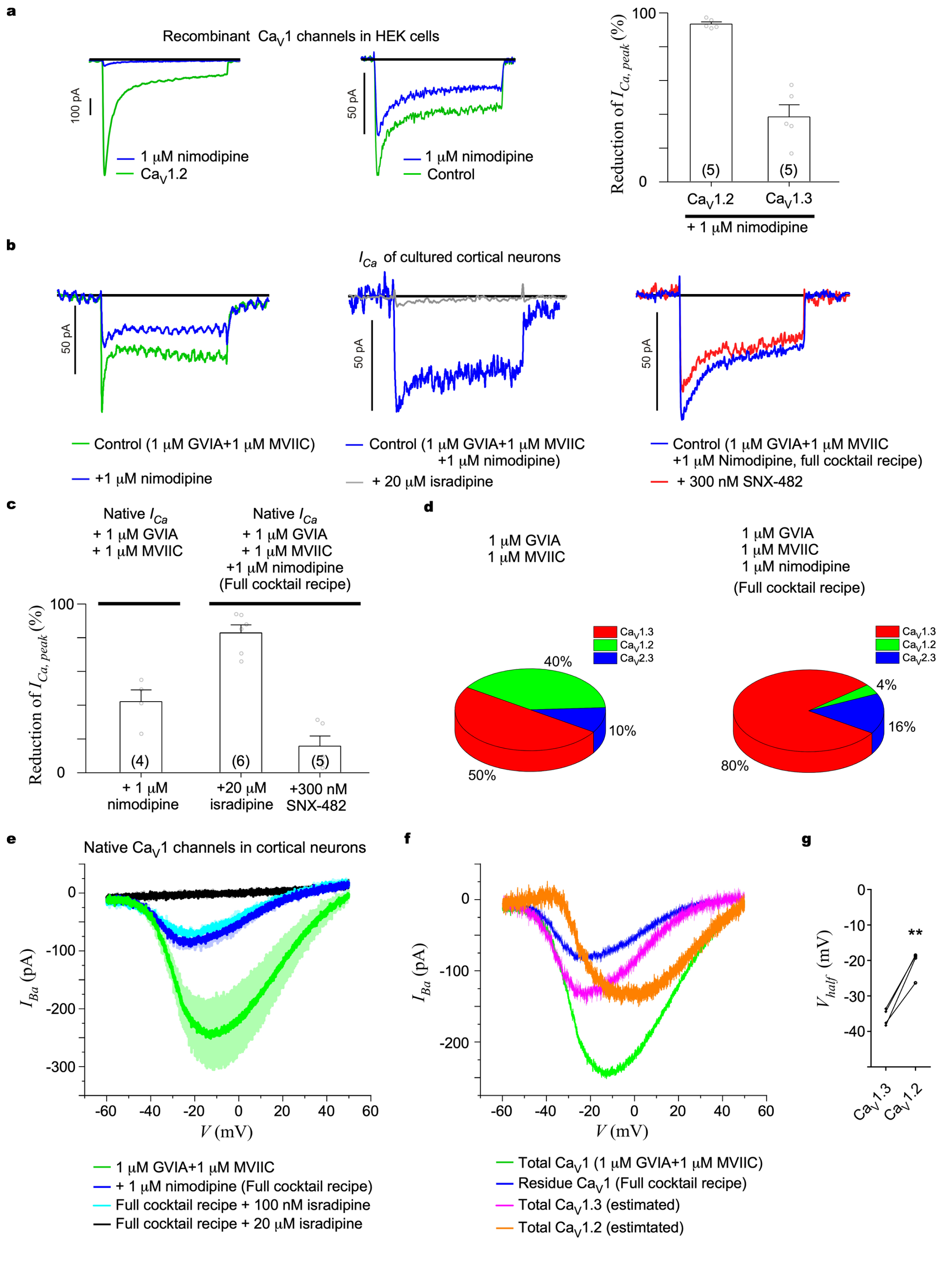

Supplementary Figure 2. Details regarding I_{Ca} mediated by Ca$_V$1 channels in cortical neurons.
a Differential blockage on recombinant Ca$_V$1.2 and Ca$_V$1.3 by 1 μM nimodipine. Evaluated at -10 mV with recombinant Ca$_V$1.2 in HEK cells, 93±1 % of the calcium current (I_{Ca}) mediated by Ca$_V$1.2 was blocked by 1 μM nimodipine, in contrast to 38±7 % for Ca$_V$1.3, confirming that 1 μM nimodipine is able to isolate Ca$_V$1.3 by blocking most Ca$_V$1.2 channels while sparing a substantial

portion of $Ca_V1.3$ channels.

b Representative native cortical I_{Ca} traces before and after applying channel inhibitors. Cultured cortical neurons were pretreated in 1 μM ω-conotoxin GVIA and 1 μM ω-conotoxin MVIIC to inhibit $Ca_V2.1$ and $Ca_V2.2$ currents, then acutely blocked by 1 μM nimodipine to isolate $Ca_V1.3$ currents (left). To verify the components of the residue currents in full-cocktail treated neurons (1 μM ω-conotoxin GVIA, 1 μM ω-conotoxin MVIIC and 1 μM nimodipine), neurons were acutely blocked by 20 μM isradipine (Ca_V1 antagonist, middle) or 300 nM SNX-482 (antagonist of R-type or $Ca_V2.3$ channels, right). These blockers were acutely applied and the inhibitory effects on I_{Ca} were evaluated several minutes after application.

c Statistic summary of different antagonists of cortical Ca_V channels. At −10mV, cortical I_{Ca} treated with $Ca_V2.1$ and $Ca_V2.2$ blockers (1 μM ω-conotoxin GVIA and 1 μM ω-conotoxin MVIIC) was reduced by 42 ± 7 % (left) upon applying 1 μM nimodipine. 83 ± 5 % of cortical I_{Ca} in the full cocktail was blocked by 20 μM isradipine, whereas the residue current (16 ± 6%) was nearly eliminated by SNX-482 (a $Ca_V2.3$ blocker), suggesting that the major components of recorded I_{Ca} from full-cocktail treated cortical neurons were mediated by $Ca_V1.3$.

d Decomposition of cortical I_{Ca}. The relative contribution to I_{Ca} treated by $Ca_V2.1$ and $Ca_V2.2$ blockers was estimated as: ~50% from $Ca_V1.3$, ~40% from $Ca_V1.2$ and ~10% from $Ca_V2.3$. After further 1 μM nimodipine blockade, i.e., treated with the full cocktail, $Ca_V1.3$ became the dominant component (~80%), compared to $Ca_V2.3$ (~16%) and $Ca_V1.2$ (~4%).

e Average *I-V* curves for Ba^{2+} currents (I_{Ba}) of cortical neurons. Cortical neurons pretreated with the Ca_V2 blockers were recorded with the voltage ramp protocol (from -60 to +50 mV), by acutely adding 1 μM nimodipine, 100 nM isradipine (isolating or confirming $Ca_V1.3$) and 20 μM isradipine (to wipe out all Ca_V1 currents). *I-V* curves from four neurons were recorded and summarized.

f Total cortical $Ca_V1.3$ versus $Ca_V1.2$ currents by estimations. The representative I_{Ba} recordings from one neuron are to demonstrate the total Ca_V1 currents (1 μM ω-conotoxin GVIA and 1 μM ω-conotoxin MVIIC, in green) and the residue Ca_V1 or $Ca_V1.3$ currents (+1 μM nimodipine, full cocktail, in blue). The $Ca_V1.3$ currents in total (pink) were estimated according to the potency of 1 μM nimodipine on $Ca_V1.3$ (62% remained, **a**). The total $Ca_V1.2$ currents (orange) were then estimated by subtracting the total $Ca_V1.3$ from the total Ca_V1 currents. Comparing the total $Ca_V1.3$

(pink) versus $Ca_V1.2$ (orange) currents resulted from the above measurements and estimations, a left-ward shift was clearly evidenced from this exemplar.

g Statistic summary of the half-activation voltage (V_{half}) for the estimated $Ca_V1.3$ and $Ca_V1.2$ channels in cortical neurons. After fitting with Boltzmann-based equation for each *I-V* curve, $Ca_V1.3$ resulted into significantly more negative V_{half} than $Ca_V1.2$ (paired Student's *t*-test: **, $p<0.01$).

Values are represented as mean±SEM.

Related to Fig. 1.

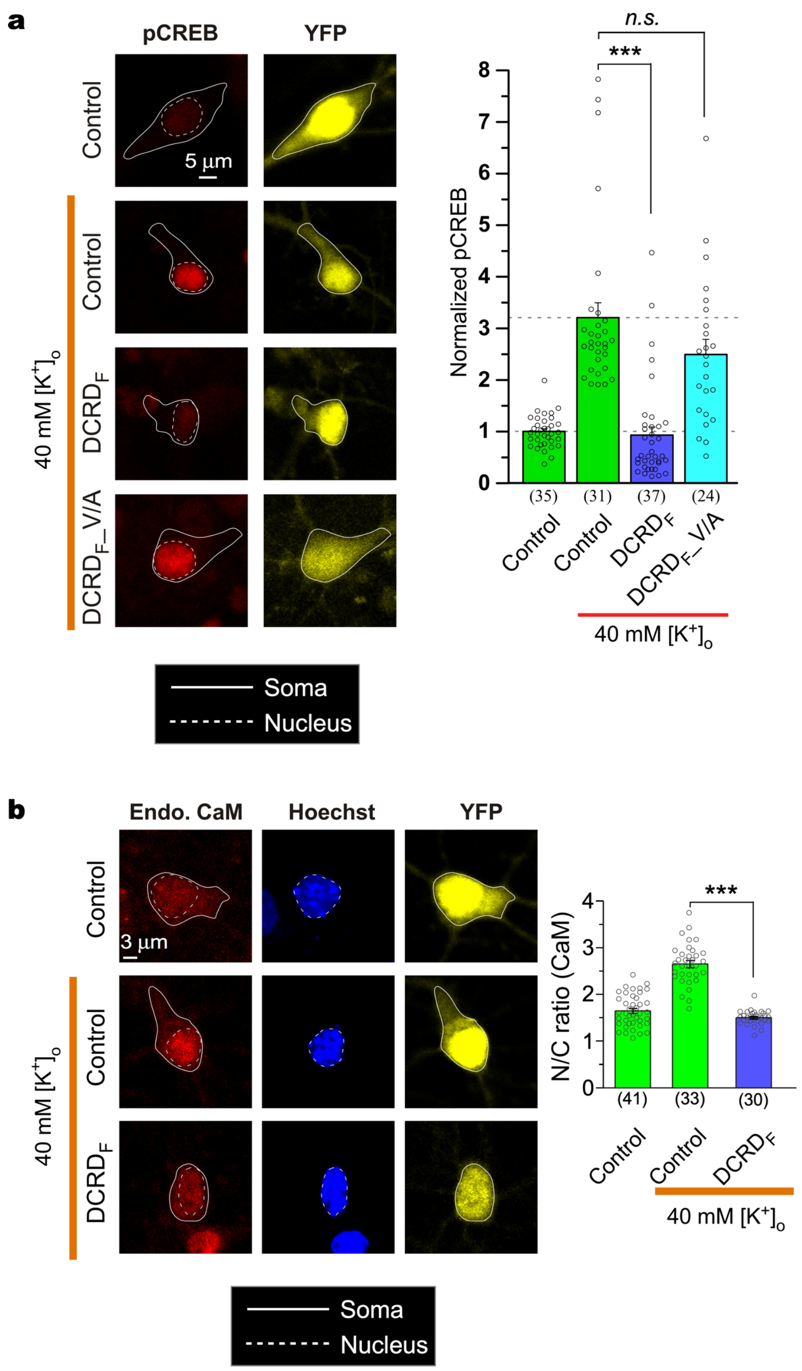

Supplementary Figure 3. DCRD$_F$ effects on Ca$_V$1-mediated neuritogenesis signals in stimulated cortical neurons.

a Effects of DCRD$_F$ peptides on pCREB signals of stimulated neurons. Cortical neurons were transfected with YFP, YFP-DCRD$_F$ or YFP-DCRD$_F$_V/A (loss-of-function mutant), respectively. Cells in TTX for at least 6 hours were stimulated by high $[K^+]_o$ for 30 min before fixation to evaluate the changes of pCREB immunofluorescence. Red and yellow fluorescence in confocal images

represent pCREB signals and overexpressed YFP, respectively. pCREB signals were normalized over the YFP control group before stimulation.

b CaM translocation in response to 40 mM $[K^+]_o$ was regulated by $DCRD_F$ peptides. N/C (nucleus/cytosol) ratio of endogenous CaM was calculated by fluorescence intensity (red) in confocal images from CaM immunostaining. Dotted and solid lines represent the envelopes of nuclei stained by Hoechst 33342 and the cell body, respectively.

Student's *t*-test (**a**), one-way ANOVA followed by Bonferroni for post hoc tests (***, $p<0.001$; *n.s.*, not significant, $p>0.05$). Values are represented as mean±SEM.

Related to Fig. 1.

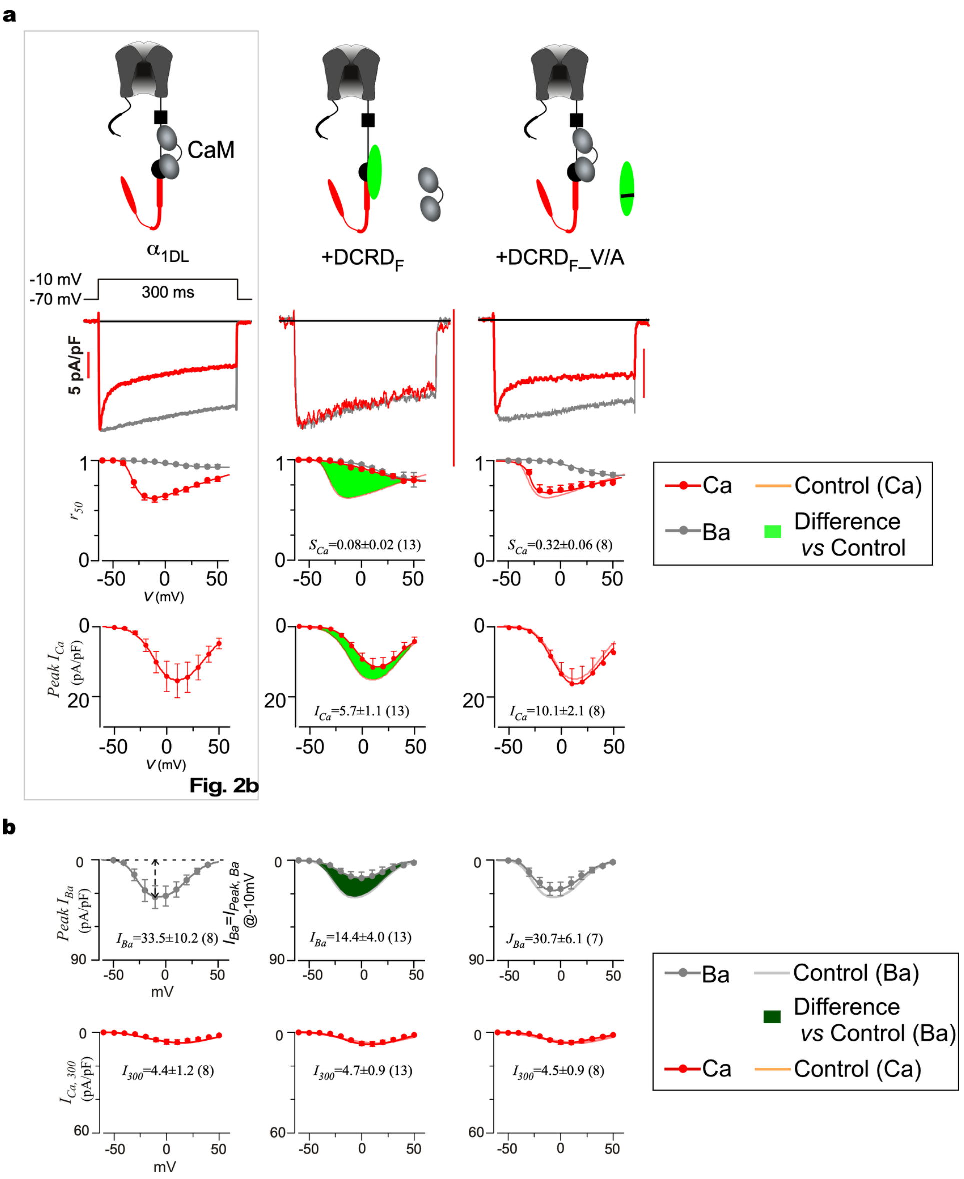

Supplementary Figure 4. DCRD$_F$ modulation of recombinant Ca$_V$1.3 channels.

a Effects of the DCRD$_F$ and DCRD$_F$_V/A on Ca$_V$1.3 (α_{1DL}) channels expressed in HEK293 cells. In the same style demonstrated by Ca$_V$1.3 control of **Fig. 2b**, from the top to the bottom row, voltage protocol, exemplary Ca^{2+} current (trace with scale bar, red) and Ba^{2+} current (rescaled to Ca^{2+} current at the peaks, gray), inactivation (r_{50}) and activation (in pA/pF, I_{Peak}) are shown respectively. The DCRD$_F$ (middle column) and the DCRD$_F$_V/A (right column) are compared with α_{1DL} control (left column) for their inactivation and activation profiles (lines in orange indicate the control). Green areas are to highlight peptide effects.

b Additional evaluations of peptide effects. Voltage-dependent action profiles for peak Ba^{2+} currents from DCRD$_F$, DCRD$_F$_V/A and α_{1DL} control group (**a**). DCRD$_F$ peptides significantly

reduced I_{Ba} of α_{1DL}, in comparison to the negative control group DCRD$_F$_V/A. Profiles of steady-state current (300 ms Ca^{2+} current, $I_{Ca,300}$) for three groups (bottom). No significant difference among three groups.

Values are represented as mean ± SEM.

Related to Fig. 2.

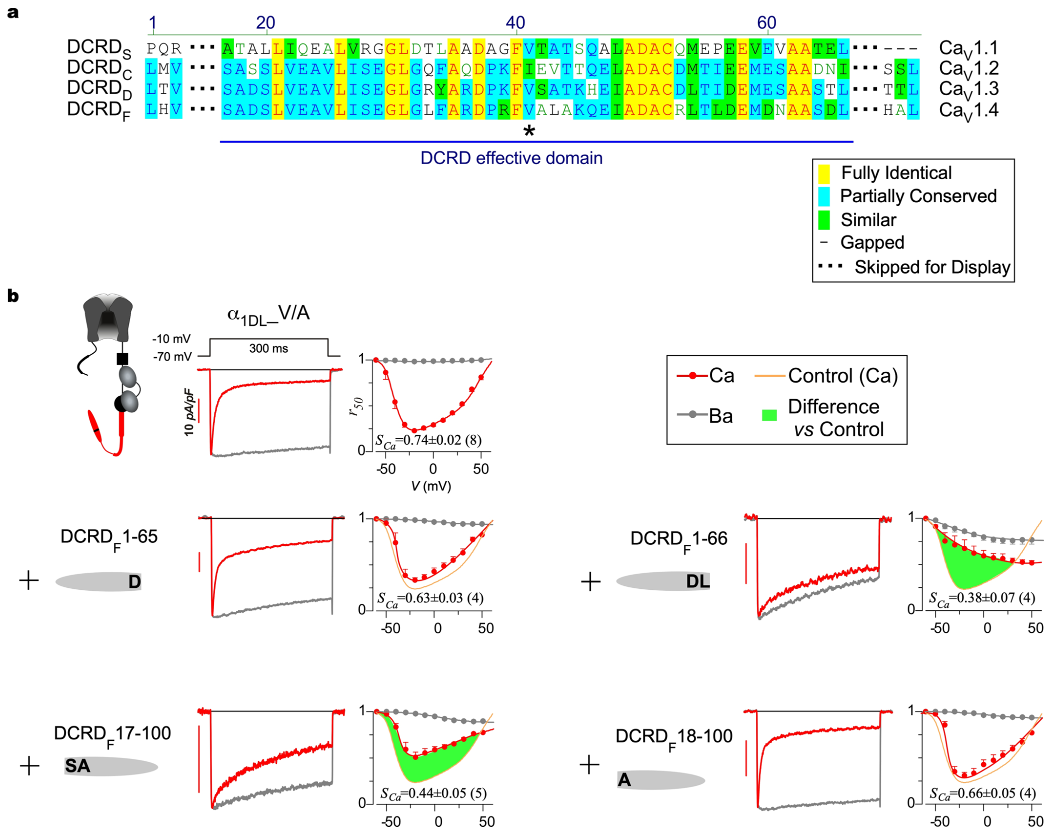

Supplementary Figure 5. Identification of the essential motif within DCRD$_F$.

a Alignment of homologous DCRD subdomains across the Ca$_V$1 family.

b α_{1DL}_V/A, which contains a loss-of-function mutation in DCRD domain[8], exhibited ultrastrong inactivation (strong Ca^{2+}-dependent inactivation or weak CMI). In the context of α_{1DL}_V/A channels and DCRD$_F$ peptides, the screening was conducted for the core DCRD motif. The fragments of DCRD$_F$1-66 and DCRD$_F$17-100 strongly attenuated Ca^{2+}-dependent inactivation of α_{1DL}_V/A (by way of peptide CMI), whereas the fragments of DCRD$_F$1-65 and DCRD$_F$18-100 exhibited rather weak CMI, highlighting the effective domain as DCRD$_F$17-66, potentially applicable to other DCRD family members.

Data are represented as mean ± SEM.

Related to Fig. 2.

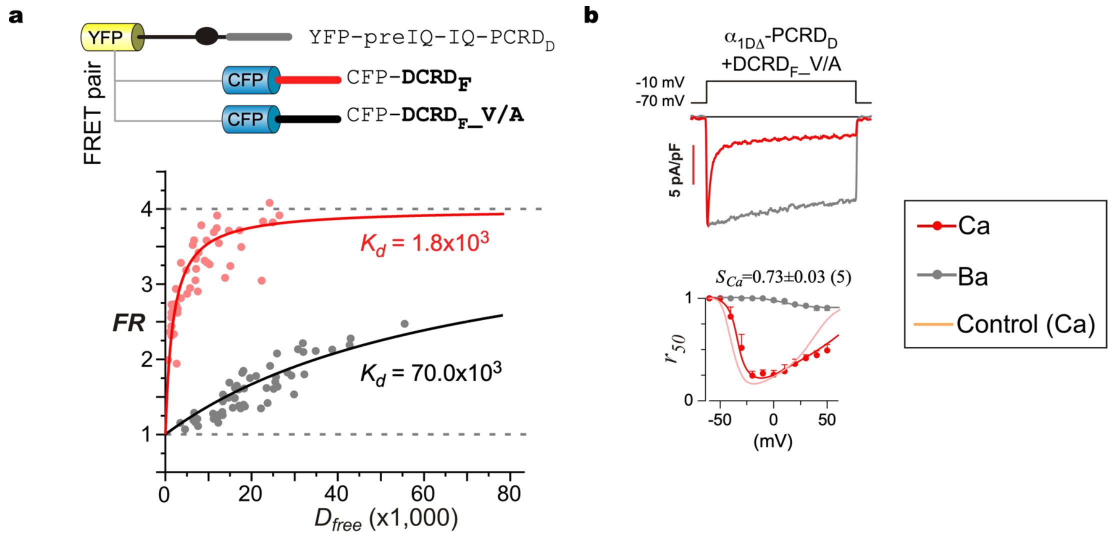

Supplementary Figure 6. DCRD$_F$_V/A attenuates both the binding affinity and the potency of inhibition.

a Binding curve between the mutant peptide CFP-DCRD$_F$_V/A and YFP-preIQ$_3$-IQ$_D$-PCRD$_D$ (black curve) by 2-hybrid 3-cube FRET. The binding curve (red) between WT CFP-DCRD$_F$ and YFP-preIQ$_3$-IQ$_D$-PCRD$_D$ was set as the reference.

b Representative traces and r_{50} profiles for $\alpha_{1D\Delta}$-PCRD$_D$ co-expressed with DCRD$_F$_V/A (red) or $\alpha_{1D\Delta}$-PCRD$_D$ alone (orange). Data are represented as mean ± SEM.

Related to Fig. 4.

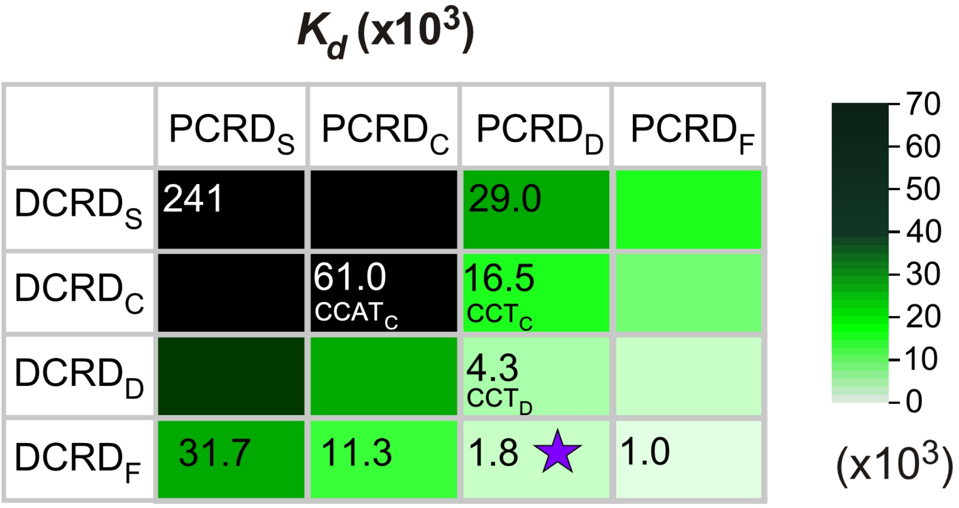

Supplementary Figure 7. Summary of K_d values for PCRD$_X$-DCRD$_X$ peptide variants.

The actual K_d values (indicated with fluorescence intensities in a.u.) are based on FRET experiments (e.g., the key peptide of P$_D$-D$_F$ labelled with the asterisk as in **Fig. 4c, f**), whereas the rest are predicted by extrapolations. The strength of affinity (K_d) is illustrated by a gradient of colors (color bar, right). Three peptides representing the native forms have additional labels of CCAT$_C$, CCT$_C$, and CCT$_D$.

Related to Fig. 4.

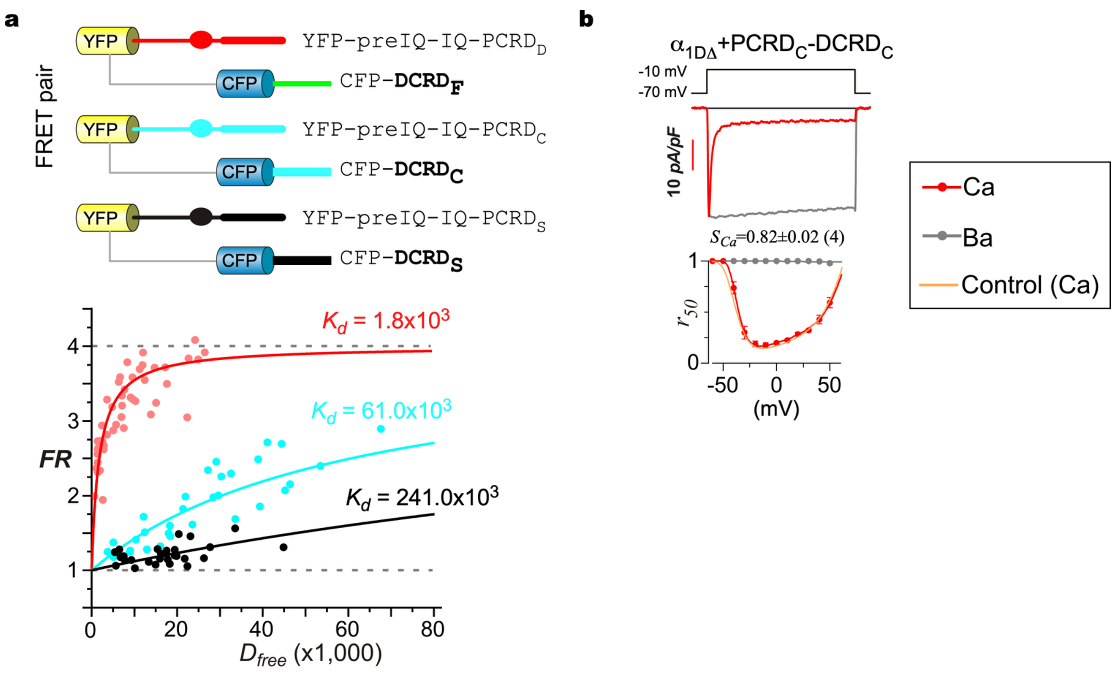

Supplementary Figure 8. FRET and electrophysiology for PCRD and DCRD variants from Ca_V1.1 and Ca_V1.2.

a Binding curves for CFP-$DCRD_C$ to YFP-$preIQ_3$-IQ_D-$PCRD_C$ (cyan) and CFP-$DCRD_S$ to YFP-$preIQ_3$-IQ_D-$PCRD_S$ (black) by 2-hybrid 3-cube FRET. FRET binding between CFP-$DCRD_F$ and YFP-$preIQ_3$-IQ_D-$PCRD_D$ (P_D/D_F) as the reference (red).

b Representative Ca^{2+} traces and inactivation (r_{50}) profiles for $\alpha_{1D\Delta}$ with $PCRD_C$-$DCRD_C$ (red), in comparison with $\alpha_{1D\Delta}$ control (line in orange).

Data are represented as mean ± SEM.

Related to Fig. 4.

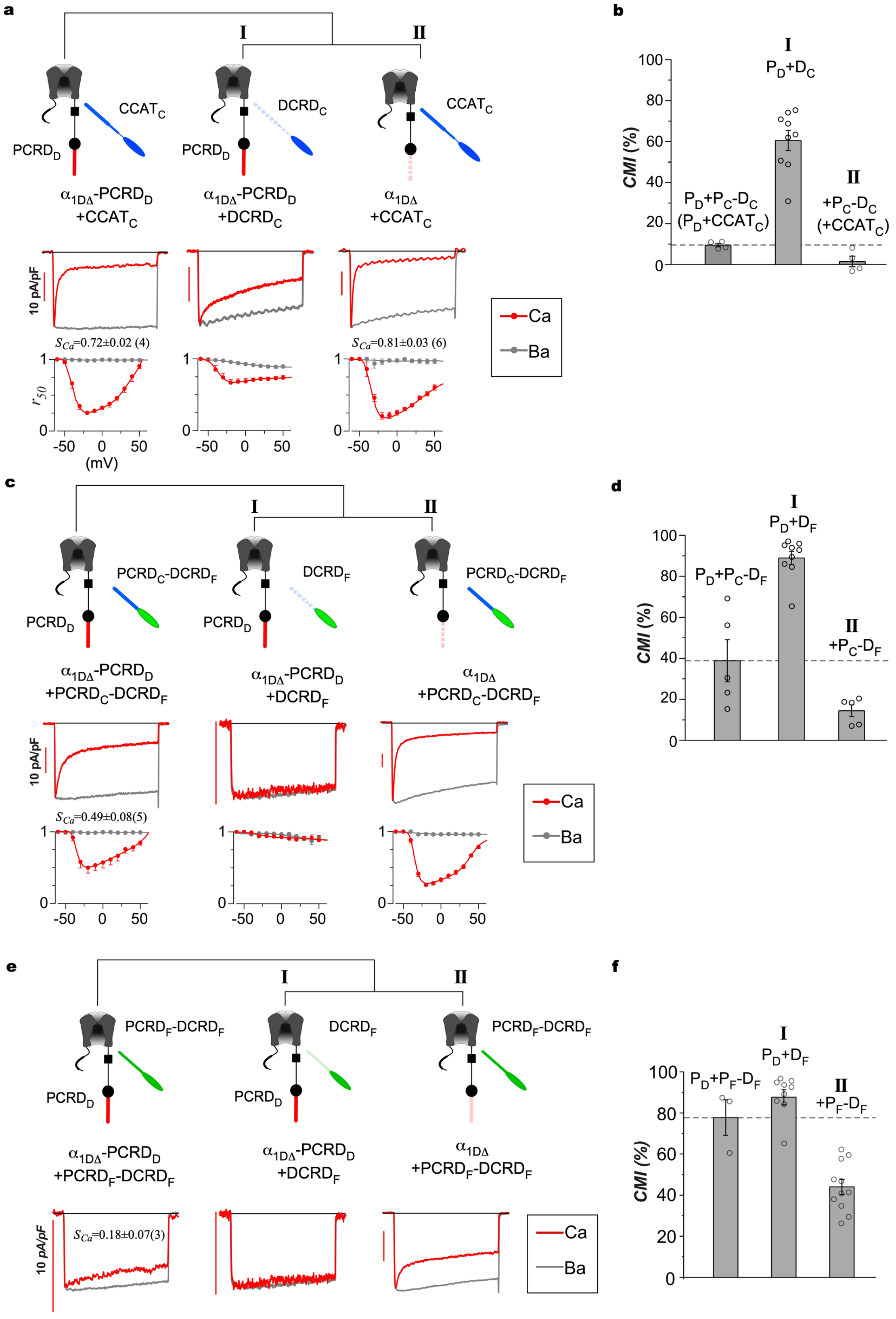

Supplementary Figure 9. Compound effects of long DCT peptides on $Ca_V1.3$ channels.

a Decomposition of $CCAT_C$ effects on $\alpha_{1D\Delta}$-$PCRD_D$ channels. CMI effects of $CCAT_C$ could be

divided into two components (top row). The first component (I) represents CMI of $DCRD_C$ (contained in $CCAT_C$) on $\alpha_{1D\Delta}$-$PCRD_D$, where PCRD and DCRD are contributed by the combination of $\alpha_{1D\Delta}$-$PCRD_D$ and $CCAT_C$, respectively. The data of component I are adopted from **Fig. 4**; and the data of component II are nearly identical to **Supplementary Figure 8** since the minor difference (in *CMI*) exists between $CCAT_C$ and $PCRD_C$-$DCRD_C$. The second component (II) represents CMI of $CCAT_C$ on $\alpha_{1D\Delta}$, where PCRD and DCRD are from the same molecule $CCAT_C$. Ca^{2+} trace exemplars (middle row) and inactivation profiles (bottom row) are shown in each column.

b Summary of CMI potency for $CCAT_C$ and its two components. The compound effects (dash line) of $\alpha_{1D\Delta}$-$PCRD_D$+$PCRD_C$-$DCRD_C$ (P_D+P_C-D_C) can be decomposed into component I ($\alpha_{1D\Delta}$-P_D+D_C) and component II ($\alpha_{1D\Delta}$+P_C-D_C).

c Decomposition of peptide $PCRD_C$-$DCRD_F$ effects on $\alpha_{1D\Delta}$-$PCRD_D$ channels in similar fashion to (**a**). CMI effects of the DCT peptide are decomposed into two components by $DCRD_C$ on $\alpha_{1D\Delta}$-$PCRD_D$ (I) and by $PCRD_C$-$DCRD_F$ on $\alpha_{1D\Delta}$ (II) (top row). Data of component I and II are adopted from **Fig. 4b** and **Fig. 4e**, respectively.

d Summary of CMI potency for $CCAT_C$ and $PCRD_C$-$DCRD_F$ and the two components for each peptide. Compound *CMI* of P_D+P_C-D_F (dash line) can be decomposed into component I (P_D+D_F) and component II (+P_C-D_F).

e Decomposition of $PCRD_F$-$DCRD_F$ effects on $\alpha_{1D\Delta}$-$PCRD_D$ channels. The first component (I) is the combination of peptide $DCRD_F$ and channel $\alpha_{1D\Delta}$-$PCRD_D$; and the second component (II) is the combination of peptide $PCRD_F$-$DCRD_F$ and channel $\alpha_{1D\Delta}$ (upper row). Current traces for P_F-D_F on $\alpha_{1D\Delta}$-$PCRD_D$ (bottom, left) and its two components (adopted from **Fig. 4b** and **Fig. 4e**, respectively).

f Statistic summary of CMI potency. The dashed line is to compare the compound effects of $\alpha_{1D\Delta}$-P_D+P_F-D_F with the two components: I, $\alpha_{1D\Delta}$-P_D+D_F (or P_D+D_F); and II, $\alpha_{1D\Delta}$+P_F-D_F (or +P_F-D_F). Data are represented as mean $\pm$ SEM.

Related to Fig. 4.

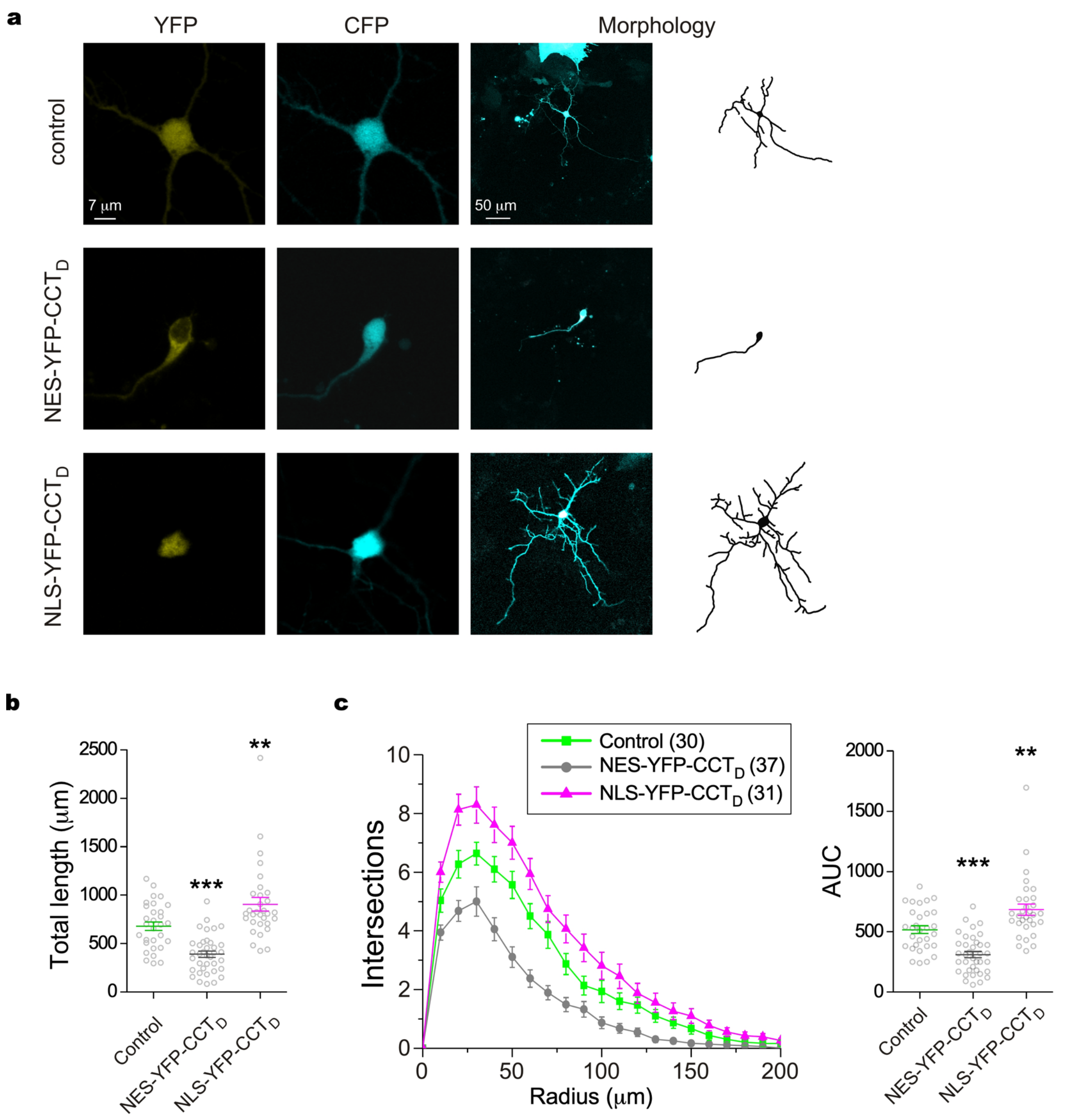

Supplementary Figure 10. DCT effects on mature neurons demonstrated by NES- or NLS-tagged CCT_D.

a Representative confocal images and neurite tracings for cortical neurons (DIV 15-18) expressing CFP (to aid tracing) and YFP-tagged CCT_D or YFP (control), respectively. YFP-CCT_D was also tagged with either NES or NLS at its N-terminus.

b, **c** Total neurite lengths (**b**) and Sholl analyses (**c**) are summarized and compared.

One-way ANOVA followed by Dunnett for post hoc test was used (**, p<0.01; ***, p<0.001). Data are represented as mean ± SEM.

Related to Fig 5.

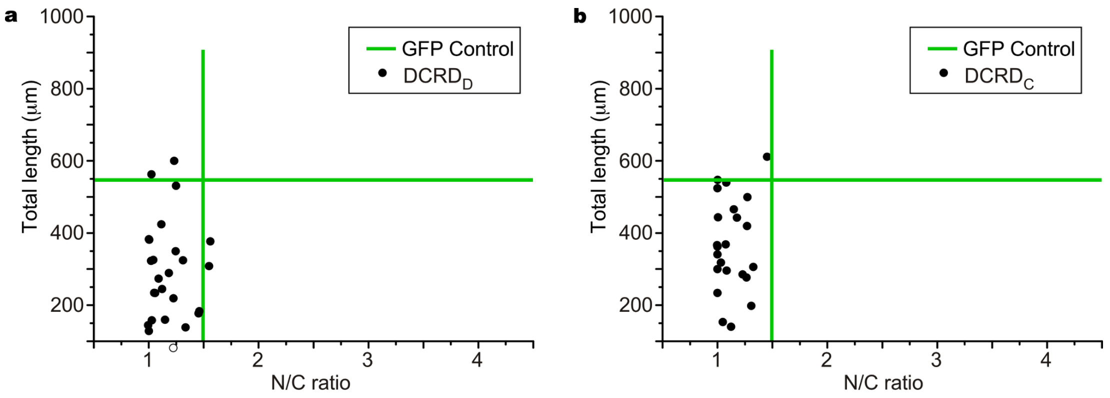

Supplementary Figure 11. Cytosolic-nuclear distribution of DCRD in relation with total neurite length.

a, **b** Scatter plots for N/C ratio of DCRD$_D$ (**a**) or DCRD$_C$ (**b**) correlated with total neurite length in cortical neurons. Horizontal and vertical lines (green) represent the GFP control.

Related to Fig. 5.

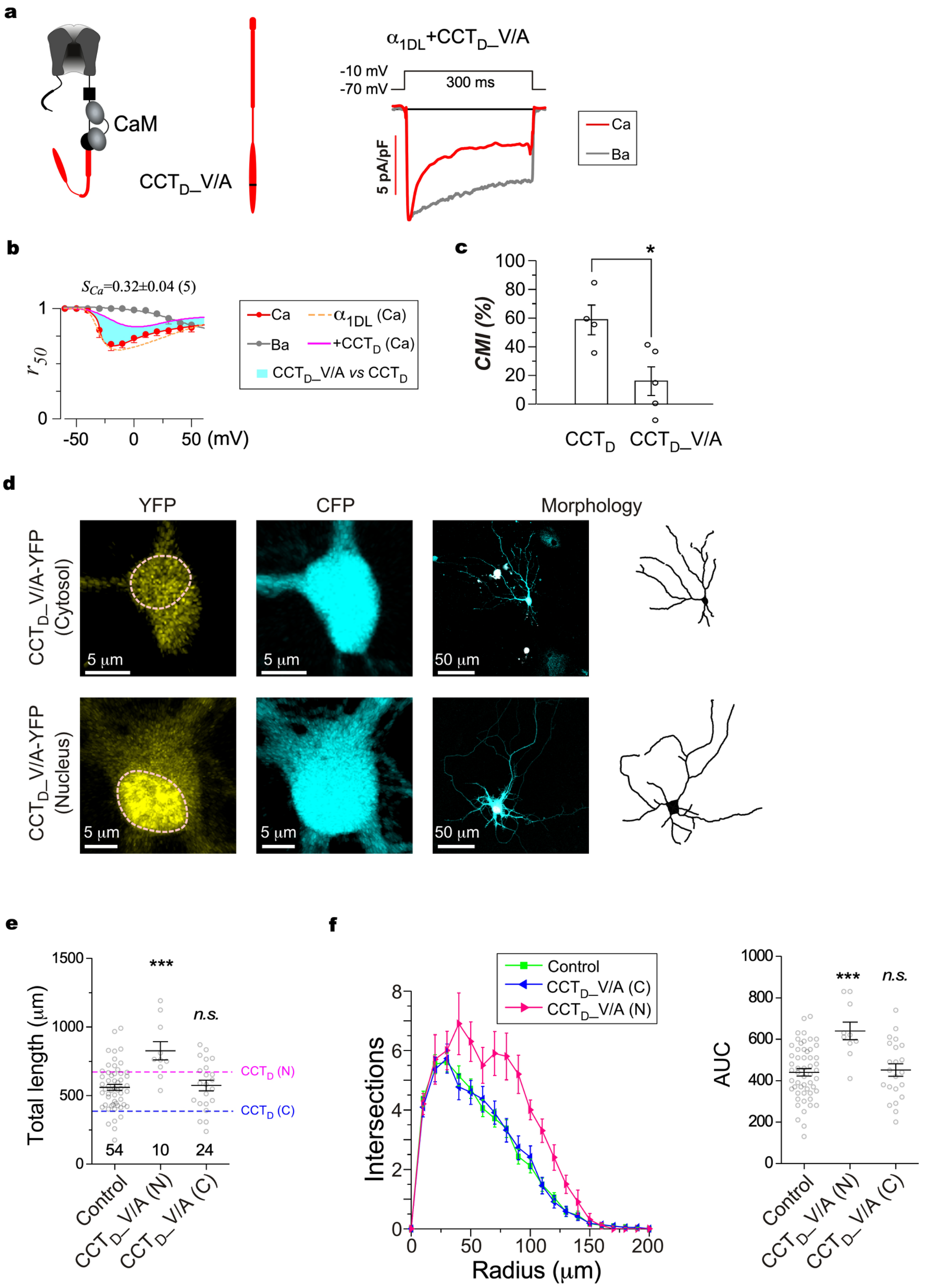

Supplementary Figure 12. Critical roles of CMI confirmed with CCT$_D$_V/A mutant in neurons. **a-c** Cartoon illustration and representative current traces for CCT$_D$_V/A effects on α_{1DL} (**a**), and on inactivation profiles (**b**) and CMI potency (**c**). In comparison with CCT$_D$_V/A (red), the solid line (in purple) and the dotted line (in orange) represent CCT$_D$ and the control group (α_{1DL}) (both adopted

from **Fig. 2**), respectively.

d-f Morphology of cortical neurons with cyto-nuclear CCT_D_V/A. By similar criteria: nuclear (N/C ratio>1.5) and cytosolic (N/C ratio<1.5), CCT_D neurons were classified into the two major groups of CCT_D (N) and CCT_D (C) respectively, demonstrated here with the confocal images and neurite tracing (**d**), analyzed by total length (**e**) or Sholl analyses (**f**) for nuclear and cytosolic CCT_D_V/A. The dashed lines in (**e**) represent the averages of CCT_D (N) and CCT_D (C) from **Fig. 5b**.

Student's *t*-test and one-way ANOVA followed by Dunnett for post hoc test were used for (**c**) and (**e**), respectively (*, $p<0.05$; **, $p<0.01$). Data are represented as mean±SEM.

Related to Fig. 5.

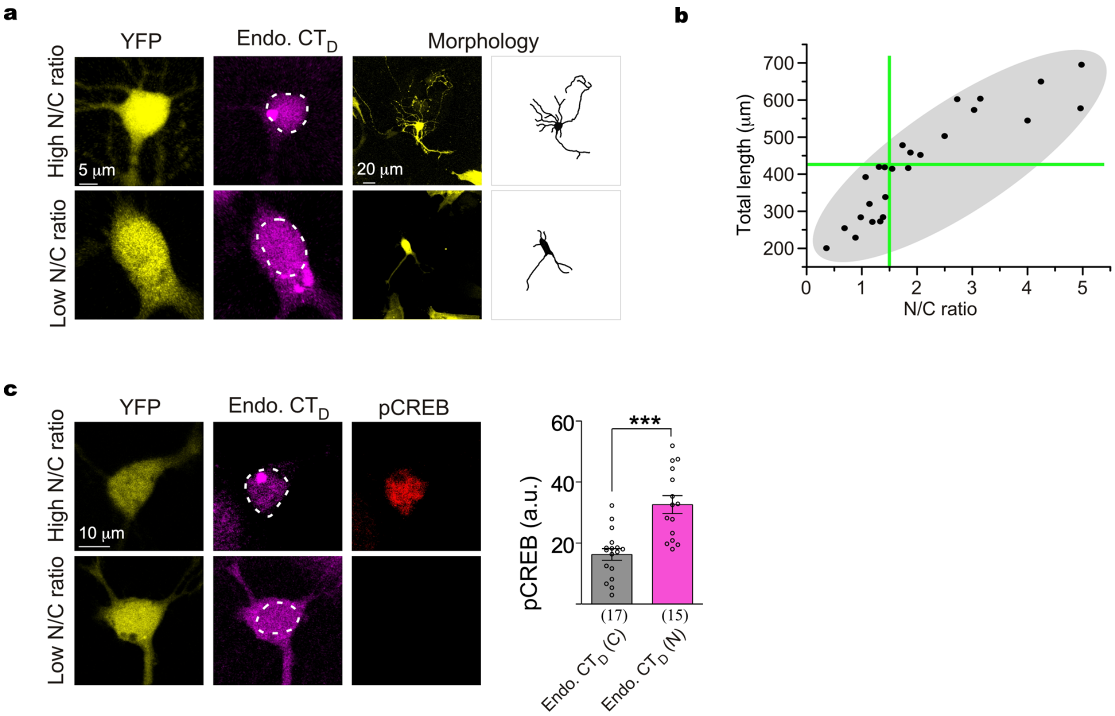

Supplementary Figure 13. Evidence regarding endogenous $Ca_V1.3$-encoded peptides in cortical neurons.

a, **b** Endogenous CTD regulated neurite outgrowth of cortical neurons in accordance to its cytosolic-nuclear distribution. By N/C ratio of the immunostaining fluorescence of endogenous CTD, neurons were categorized into two subgroups (high N/C ratio versus low N/C ratio), respectively (**a**). Subcellular distribution of CTD was correlated with the total neurite length (**b**), analyzed in a similar style to **Fig. 5i**.

c Differential effects on pCREB by cytosolic versus nuclear CTD peptides endogenous to cortical neurons. Confocal images (left) illustrate YFP (yellow, soma contour), endogenous CTD (in purple) and pCREB (in red) for neurons with high (top) or low (bottom) N/C ratio of endogenous CTD. The dashed circles indicate the envelope of nuclei stained by Hoechst. Fluorescence intensities of nuclear pCREB are compared for the cytosolic versus nuclear subgroups of neurons. Student's *t*-test was used (***, $p<0.001$). Values are represented as mean±SEM.

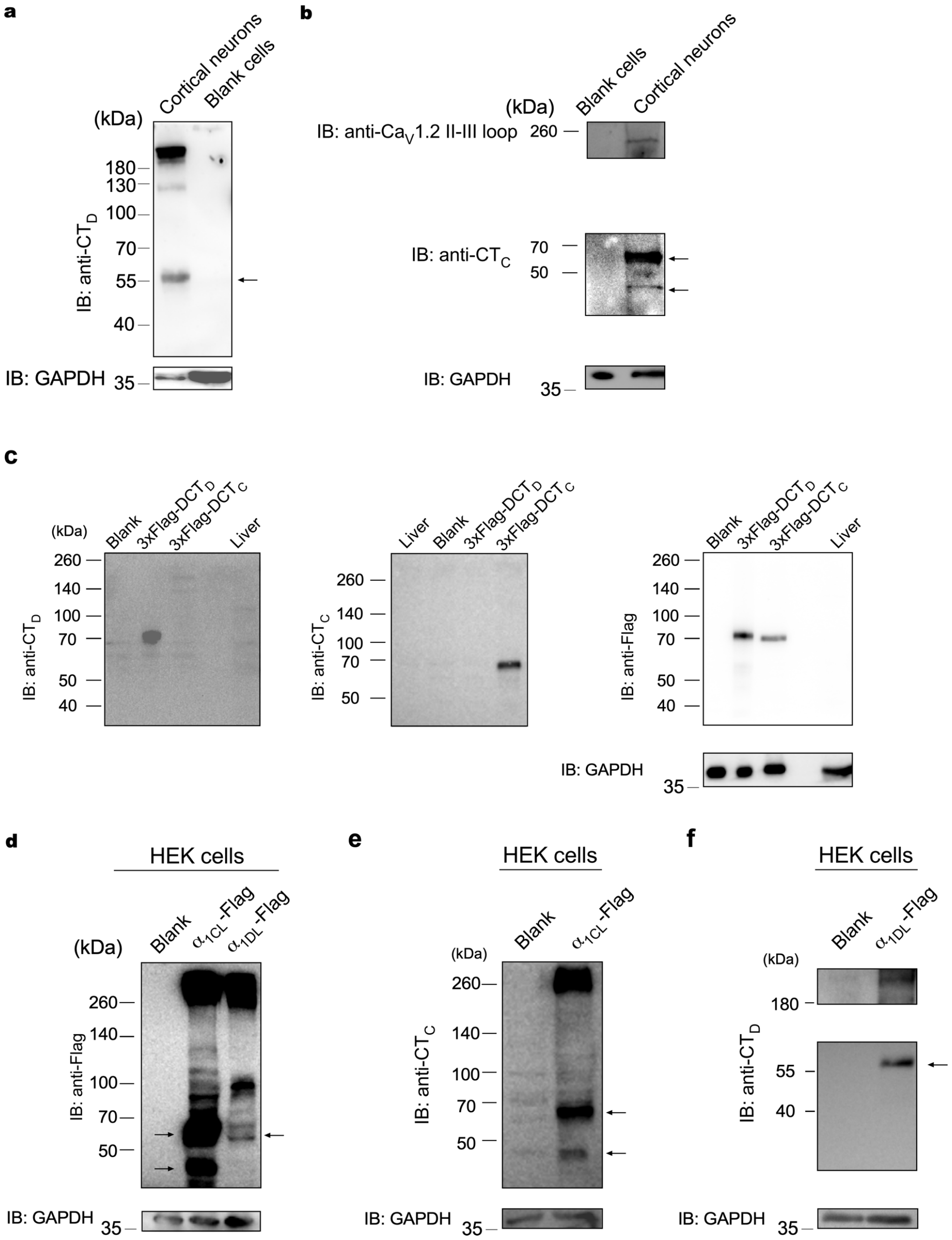

Supplementary Figure 14. Western blots for DCT peptides encoded by $Ca_V1.3$ versus $Ca_V1.2$. **a** Endogenous CT_D peptides in cortical neurons. Western-blot analyses were conducted for cortical neurons of newborn mice and CHO blank cells. Equal amounts of proteins were subject to gel electrophoresis separately for blotting by antibodies against CT_D (C-terminus of α_{1D}, top gel) or GAPDH (bottom gel, as the input reference). CT_D immunoreactivities were detected only in cortical neurons. The bands of ~200 kDa and ~60 kDa (marked with an arrow) are supposed to represent the full-length α_{1D} channel and the CT_D fragment, respectively.

b Endogenous CT_C peptides in cortical neurons. Similar to (**a**), equal amounts of proteins were subject to gel electrophoresis separately for blotting by antibodies against $Ca_V1.2$ II-III loop (top gel), CT_C (C-terminus of α_{1D}, middle gel) or GAPDH (bottom gel, reference). The band of ~200 kDa represents the full-length α_{1C} channel, and the two bands of upper (~60 kDa) and lower (~40 kDa) (marked with arrows) represent the CT_C peptides.

c Validation of CT_C and CT_D antibodies. Western blots of blank CHO cells, CHO cells expressing 3xFlag-DCT_D or 3xFlag-DCT_C, and liver tissues from neonatal mice. Bands in the supposed size of 3xFlag-DCT_D or 3xFlag-DCT_C were detected by anti-CT_D (left), anti-CT_C (middle) or anti-Flag (left), further confirming antibody specificity. Equal amounts of proteins were subject to gel electrophoreses separately for blotting by antibodies against CT_D (left), CT_C (middle), Flag (top right) or GAPDH (bottom right, reference).

d Ca_V1-encoded peptides generated in HEK cells. HEK cells were expressed with α_{1DL}-Flag or α_{1CL}-Flag, analyzed by anti-Flag (top gel) and anti-GAPDH (bottom gel, reference) antibodies. The upper band supposedly from the full-length α_{1C} was detected, together with the two lower bands of ~60 kDa and ~40 kDa. In comparison, besides a similar full-size band of α_{1D}, a ~60 kDa band was detected from α_{1DL}-Flag overexpressing in HEK cells, while no band around 40 kDa was detected. Blots of anti-Flag and anti-GADPH were from the same gel cropped for independent blots.

e DCT_C bands detected by anti-CT_C from recombinant $Ca_V1.2$. HEK cells expressing α_{1CL}-Flag resulted in two bands, i.e., an upper (~60 kDa) band and a lower (~40 kDa) band. Blots of anti-CT_C and anti-GADPH (reference) were from the same gel cropped for independent blots.

f DCT_D bands detected by anti-CT_D from recombinant $Ca_V1.3$. HEK cells expressing α_{1DL}-Flag resulted in one single band of ~60 kDa. Equal amounts of proteins were subject to gel electrophoreses separately for blotting by antibodies against CT_D (top and middle subpanels) or GAPDH (bottom, reference). The two anti-CT_D bands were from the same gel cropped for separate exposure.

Related to Fig. 6.

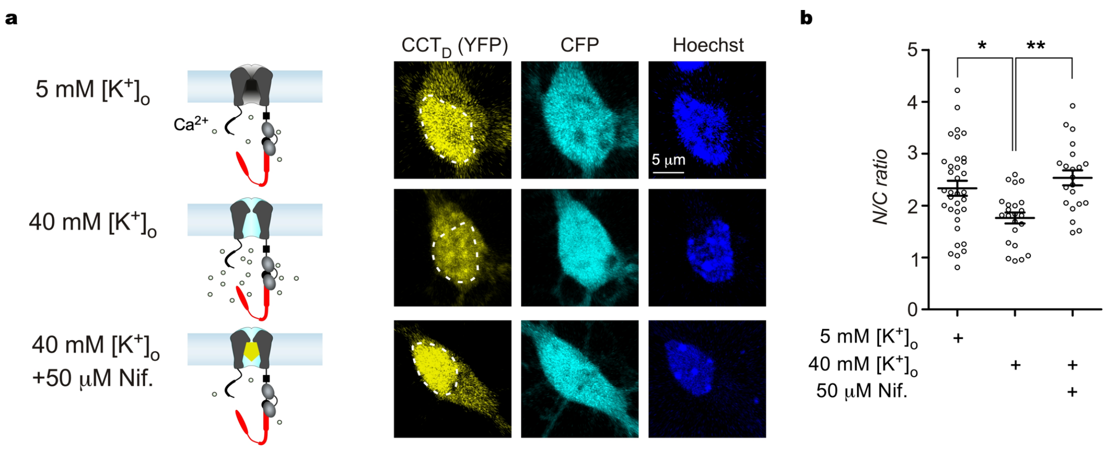

Supplementary Figure 15. Cytosolic-nuclear translocation of DCT peptides is sensitive to Ca$_V$1 influx.

a Illustration of Ca$_V$1 under different conditions (left). Cortical neurons were maintained in 5 mM [K^+]$_o$ solutions as the basal condition (control, top), and then stimulated with 40 mM [K^+]$_o$ to induce Ca^{2+} influx (stimulated, middle). 50 μM nifedipine (Nif.) blocked Ca$_V$1 under the conditions of 40 mM [K^+]$_o$ (blocked, bottom). For each condition, confocal images represent YFP (CCT$_D$ distribution), CFP (soma contour) and Hoechst (nuclear envelop), respectively (right).

b Cytonuclear translocations indexed with N/C ratio values. N/C ratio of the stimulation group was significantly suppressed when compared with the control group; whereas blockage of Ca$_V$1 channels eliminated such change in N/C ratio. One-way ANOVA followed by Bonferroni for post hoc tests were used (*, p<0.05; **, p<0.01). Data are represented as mean±SEM.

Related to Fig. 7.

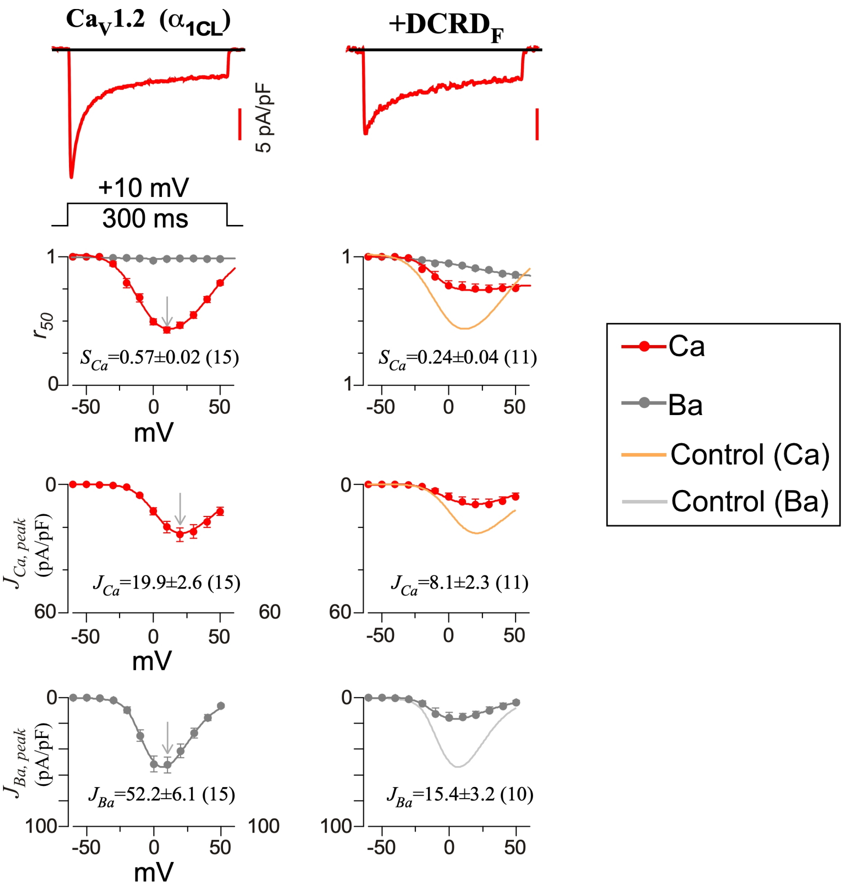

Supplementary Figure 16. Peptide CMI is expandable to $Ca_V1.2$ channels.

The voltage protocol, exemplary Ca^{2+} currents (traces with scale bars, red), and inactivation (r_{50}) and activation (in pA/pF, J_{peak} in Ca^{2+} or Ba^{2+}) are shown respectively for $Ca_V1.2$ channels expressed in HEK cells with (left) or without (right) $DCRD_F$ peptides. Notably, the activation voltage of $Ca_V1.2$ is positively shifted as compared to $Ca_V1.3$ (**Fig. 2b**), known as one major difference between these two channel subtypes[9], which is considered to underlie the critical importance of $Ca_V1.3$ in neuronal signaling[10]. In addition, CMI effects on $Ca_V1.2$ were less pronounced than $Ca_V1.3$ even with the ultrastrong $DCRD_F$ (**Supplementary Figure 4**), supposedly due to the weak $PCRD_C$ of $Ca_V1.2$ (**Fig. 4**).

Values are represented as mean±SEM.

Related to Fig. 8.

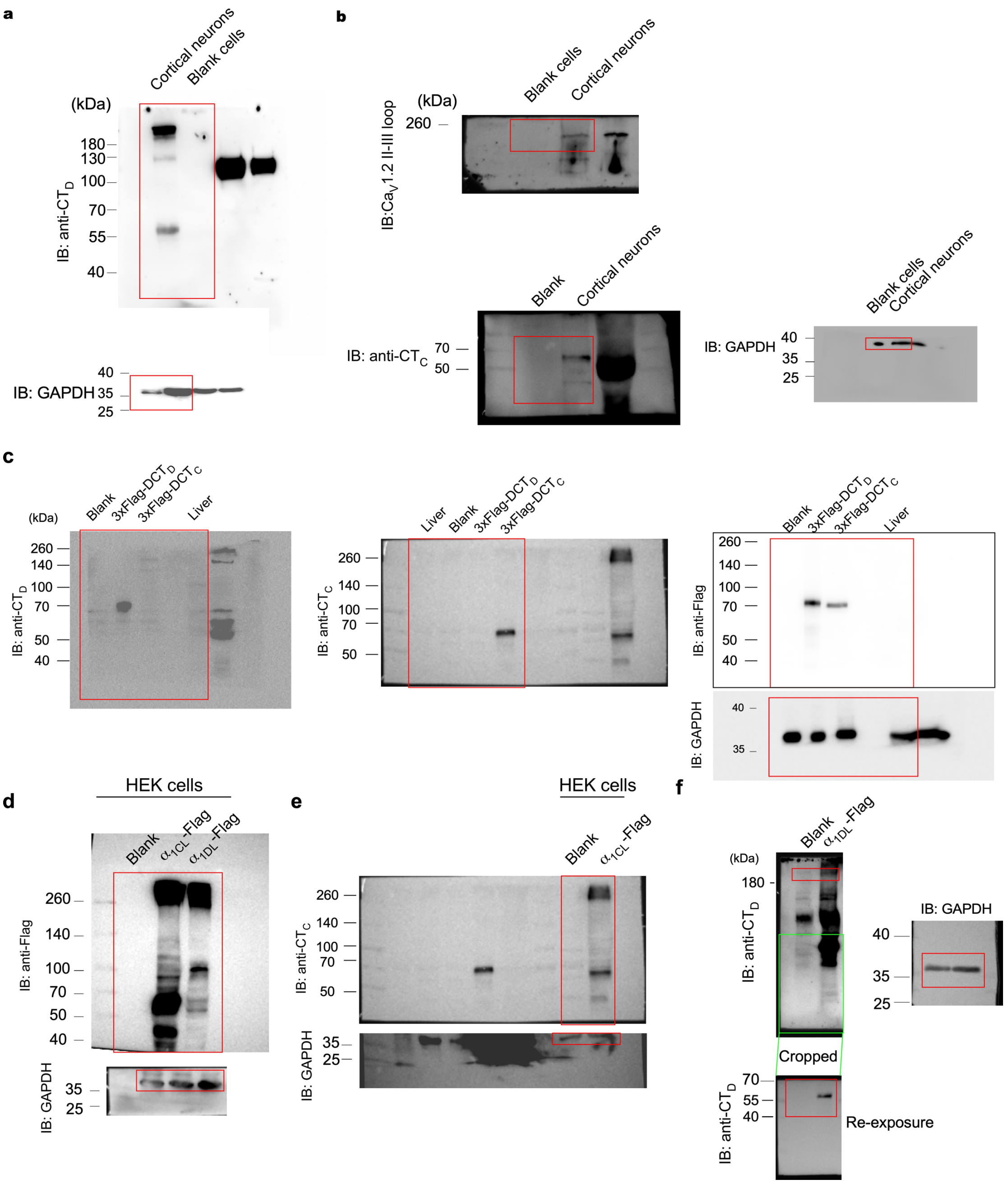

Supplementary Figure 17. Uncropped blots.

a Blots in Supplementary Figure 14a.

b Blots in Supplementary Figure 14b.

c Blots in Supplementary Figure 14c.

d Blots in Supplementary Figure 14d.

e Blots in Supplementary Figure 14e.

f Blots in Supplementary Figure 14f.

Abbreviations	Coding/Meaning	Alternative Names	Notes
DCT	Distal carboxyl terminus		The full-length peptide or motif
$PCRD_X$	Proximal domain of DCT	P_X	Subscription X=S, C, D or F
$DCRD_X$	Distal domain of DCT	D_X	X=S, C, D or F
DCT_X	DCT variants		X=S, C, D or F
$CCAT_C$	Calcium channel associated transcription regulator, encoded by $Ca_V1.2$ DCT	DCT_C	Nearly identical to DCT_C
CCT_X	Cleaved carboxyl-terminal fragment		X=C or D; functionally similar to $DCRD_X$
CT_D	Native peptides encoded by the carboxyl terminus of $Ca_V1.3$		~60 kDa; functionally resembling DCT_D and CCT_D
CT_C	Native peptides encoded by the carboxyl terminus of $Ca_V1.2$		~60 kDa and ~40 kDa, presumably corresponding to $CCAT_C$ and CCT_C, respectively

Supplementary Table 1. Summary of the abbreviations for the names of Ca_V1-encoded peptides.

Supplementary References

1. Gomez-Ospina N, Tsuruta F, Barreto-Chang O, Hu L, Dolmetsch R. The C terminus of the L-type voltage-gated calcium channel Ca(V)1.2 encodes a transcription factor. *Cell* **127**, 591-606 (2006).

2. Schroder E, Byse M, Satin J. L-type calcium channel C terminus autoregulates transcription. *Circ Res* **104**, 1373-1381 (2009).

3. Lu L*, et al.* Regulation of gene transcription by voltage-gated L-type calcium channel, Cav1.3. *J Biol Chem* **290**, 4663-4676 (2015).

4. Gomez-Ospina N*, et al.* A promoter in the coding region of the calcium channel gene CACNA1C generates the transcription factor CCAT. *PLoS One* **8**, e60526 (2013).

5. Du X*, et al.* alpha1ACT Is Essential for Survival and Early Cerebellar Programming in a Critical Neonatal Window. *Neuron* **102**, 770-785 e777 (2019).

6. Hulme JT*, et al.* Sites of proteolytic processing and noncovalent association of the distal C-terminal domain of CaV1.1 channels in skeletal muscle. *Proc Natl Acad Sci U S A* **102**, 5274-5279 (2005).

7. Yang L, Katchman A, Samad T, Morrow J, Weinberg R, Marx SO. beta-adrenergic regulation of the L-type Ca2+ channel does not require phosphorylation of alpha1C Ser1700. *Circ Res* **113**, 871-880 (2013).

8. Liu X, Yang PS, Yang W, Yue DT. Enzyme-inhibitor-like tuning of Ca(2+) channel connectivity with calmodulin. *Nature* **463**, 968-972 (2010).

9. Xu W, Lipscombe D. Neuronal Ca(V)1.3alpha(1) L-type channels activate at relatively hyperpolarized membrane potentials and are incompletely inhibited by dihydropyridines. *J Neurosci* **21**, 5944-5951 (2001).

10. Zhang H*, et al.* Association of Ca(V)1.3 L-type calcium channels with shank. *Journal of Neuroscience* **25**, 1037-1049 (2005).
