# Supplementary material for: Cytosolic peptides encoding CaV1 C-termini downregulate the calcium channel activity-neuritogenesis coupling
Source: Commun Biol. 2022 May 19;5:484. doi: 10.1038/s42003-022-03438-1 (PMC9120191; doi:10.1038/s42003-022-03438-1)
Supplement: Supplementary file 5 — Reporting Summary [file 42003_2022_3438_MOESM5_ESM.pdf]

nature portfolio

Corresponding author(s): Xiaodong Liu

Last updated by author(s): Mar 24, 2022

Reporting Summary

Nature Portfolio wishes to improve the reproducibility of the work that we publish. This form provides structure for consistency and transparency in reporting. For further information on Nature Portfolio policies, see our Editorial Policies and the Editorial Policy Checklist.

Statistics

For all statistical analyses, confirm that the following items are present in the figure legend, table legend, main text, or Methods section.

n/a	Confirmed	
☐	☒	The exact sample size (*n*) for each experimental group/condition, given as a discrete number and unit of measurement
☐	☒	A statement on whether measurements were taken from distinct samples or whether the same sample was measured repeatedly
☐	☒	The statistical test(s) used AND whether they are one- or two-sided<br>*Only common tests should be described solely by name; describe more complex techniques in the Methods section.*
☒	☐	A description of all covariates tested
☐	☒	A description of any assumptions or corrections, such as tests of normality and adjustment for multiple comparisons
☐	☒	A full description of the statistical parameters including central tendency (e.g. means) or other basic estimates (e.g. regression coefficient) AND variation (e.g. standard deviation) or associated estimates of uncertainty (e.g. confidence intervals)
☐	☒	For null hypothesis testing, the test statistic (e.g. *F*, *t*, *r*) with confidence intervals, effect sizes, degrees of freedom and *P* value noted<br>*Give P values as exact values whenever suitable.*
☒	☐	For Bayesian analysis, information on the choice of priors and Markov chain Monte Carlo settings
☒	☐	For hierarchical and complex designs, identification of the appropriate level for tests and full reporting of outcomes
☒	☐	Estimates of effect sizes (e.g. Cohen's *d*, Pearson's *r*), indicating how they were calculated

Our web collection on statistics for biologists contains articles on many of the points above.

Software and code

Policy information about availability of computer code

Data collection	ZStudio (custom software for patch clamp recording), Zen 2009 (Zeiss), Andor iQ2 (Andor)
Data analysis	ZStudio (custom software for analyzing patch clamp data), imaris 7.7.2 (Bitplane), Fiji: imageJ (NIH), matlab R2010b (MathWorks), Origin 2019b (OriginLab), GraphPad Prism 5 (GraphPad Software)

For manuscripts utilizing custom algorithms or software that are central to the research but not yet described in published literature, software must be made available to editors and reviewers. We strongly encourage code deposition in a community repository (e.g. GitHub). See the Nature Portfolio guidelines for submitting code & software for further information.

Data

Policy information about availability of data

All manuscripts must include a data availability statement. This statement should provide the following information, where applicable:

- Accession codes, unique identifiers, or web links for publicly available datasets
- A description of any restrictions on data availability
- For clinical datasets or third party data, please ensure that the statement adheres to our policy

The data in details associated with the main figures have been deposited onto https://doi.org/10.5061/dryad.cvdncjt63. Source data for the graphs in this study can be found from Supplementary Data 1. Other data and information are available from the corresponding author upon reasonable request.

Field-specific reporting

Please select the one below that is the best fit for your research. If you are not sure, read the appropriate sections before making your selection.

☒ Life sciences ☐ Behavioural & social sciences ☐ Ecological, evolutionary & environmental sciences

For a reference copy of the document with all sections, see nature.com/documents/nr-reporting-summary-flat.pdf

Life sciences study design

All studies must disclose on these points even when the disclosure is negative.

Sample size	Patch clamp recording: n>3; neuronal imaging: n>15; FRET: n>30; western blot: n≥3. Sample sizes here provided sufficient statistic significance in same type of studies.
Data exclusions	No data from measurements that passed pre-established quality control were excluded. For patch clamp recording, the criteria include current, leak and resistance. For neuronal imaging, neurons without intact cell body were excluded. For FRET assays, cells with aberrant CFP/YFP ratio were excluded.
Replication	All data have been replicated for at least twice.
Randomization	NA
Blinding	NA

Reporting for specific materials, systems and methods

We require information from authors about some types of materials, experimental systems and methods used in many studies. Here, indicate whether each material, system or method listed is relevant to your study. If you are not sure if a list item applies to your research, read the appropriate section before selecting a response.

Materials & experimental systems

n/a	Involved in the study	
☐	☒	Antibodies
☐	☒	Eukaryotic cell lines
☒	☐	Palaeontology and archaeology
☐	☒	Animals and other organisms
☒	☐	Human research participants
☒	☐	Clinical data
☒	☐	Dual use research of concern

Methods

n/a	Involved in the study	
☒	☐	ChIP-seq
☒	☐	Flow cytometry
☒	☐	MRI-based neuroimaging

Antibodies

Antibodies used	CaM (Rabbit mAb#5197-1, Epitomics, Species Cross-Reactivity: Human, Mouse, Rat)<br>pCREB (Rabbit mAb #9198, Cell Signaling Technology, Species Cross-Reactivity: Human, Mouse, Rat)<br>c-Fos (Rabbit mAb [EPR21930-238] #ab222699, Abcam, Species Cross-Reactivity: Mouse, Human)<br>anti-CaV1.3 CT (a.a. 2025-2161) (Mouse mAb [N38/8] #ab84811, Abcam, Species Cross-Reactivity: Mouse, Rat, Rabbit, Human)<br>anti-CaV1.2 CT (a.a. 1835-2135) (Rabbit pAb #21774-1-AP, proteintech, Species Cross-Reactivity: human, mouse, rat);<br>anti-CaV1.2 II-III loop (#ACC-003, allomone, Source: Rabbit, Species Cross-Reactivity: Human, Mouse, Rat),<br>anti-Flag (#20543-1-AP, proteintech, Source: Rabbit, Species Cross-Reactivity: recombinant protein with Flag tag)<br>anti-GAPDH, (#P01L081, Gene-Protein Link, Source: Rabbit, Species Cross-Reactivity: Human, Mouse, Rat, Bovine, Pig, Chicken, Zebrafish, Green Monkey, Dilutions: 1:5000 in PBS)
Validation	CaM antibody validation: Ma 2014 Cell; Deisseroth 1998 Nature<br>pCREB antibody validation: Ma 2014 Cell; Yang 2018 Nat. Comm.<br>c-Fos antibody validation: https://www.abcam.com/nav/primary-antibodies/rabbit-monoclonal-antibodies/c-fos-antibody-epr21930-238-ab222699.html<br>anti-CaV1.3 CT antibody validation: Chovancova 2020 Biochem Pharmacol and Supplementary Figure 14<br>anti-CaV1.2 CT antibody validation: https://www.ptgcn.com/products/L-VOCC-Antibody-21774-1-AP.htm<br>anti-CaV1.2 II-III loop antibody validation: https://www.alomone.com/p/anti-cav1-2-antibody/ACC-003<br>anti-Flag antibody validation: Supplementary Figure 14<br>anti-GAPDH antibody validation: Supplementary Figure 14

Eukaryotic cell lines

Policy information about cell lines

Cell line source(s)	HEK293, CHO
Authentication	HEK293 cells were from ATCC, CHO cells were from Cell Resource Center, IBMS, CAMS/PUMC
Mycoplasma contamination	HEK293 cells (ATCC) were checked by PCR with primers 5'- GGCGAATGGGTGAGTAACACG -3' and 5'- CGGATAACGCTTGCGACCTATG -3' to ensure free of mycoplasma contamination. CHO cells were not tested for mycoplasma contamination.
Commonly misidentified lines (See ICLAC register)	NA

Animals and other organisms

Policy information about studies involving animals; ARRIVE guidelines recommended for reporting animal research

Laboratory animals	newborn ICR mice
Wild animals	NA
Field-collected samples	NA
Ethics oversight	All animals were obtained from the laboratory animal research centers, Tsinghua University and Peking University. Procedures involving animals have been approved by local institutional ethical committees of Tsinghua University and Beihang University.

Note that full information on the approval of the study protocol must also be provided in the manuscript.
